# *Bulleidia extructa* PP_925: Genome Reduction, Minimalist Metabolism, and Evolutionary Insights into Firmicutes Diversification

**DOI:** 10.3390/ijms27010448

**Published:** 2025-12-31

**Authors:** Peter V. Evseev, Irina V. Podoprigora, Andrei V. Chaplin, Zurab S. Khabadze, Artem A. Malkov, Lyudmila I. Kafarskaia, Dmitriy A. Shagin, Yulia N. Urban, Olga Yu. Borisova, Boris A. Efimov

**Affiliations:** 1 Pirogov Russian National Research Medical University, Ostrovityanova Street 1, Moscow 117997, Russia; okolomedik@gmail.com (A.V.C.); xkairyx@gmail.com (A.A.M.); likmed@mail.ru (L.I.K.); shagdim777@gmail.com (D.A.S.); 2 Institute of Medicine, Peoples’ Friendship University of Russia Named After Patrice Lumumba, Miklukho-Maklaya Street 6, Moscow 117998, Russia; anohinairina@yandex.ru (I.V.P.); khabadze_zs@pfur.ru (Z.S.K.); 3 G. N. Gabrichevsky Research Institute for Epidemiology and Microbiology, Admirala Makarova Street 10, Moscow 125212, Russia; urban@gabrich.ru (Y.N.U.); olgborisova@mail.ru (O.Y.B.)

**Keywords:** *Bulleidia extructa* PP_925, *Bulleidia extructa*, *Bulleidia*, *Bacillota*, *Erysipelotrichaceae*, *Erysipelotrichales*, *Erysipelotrichia*, *Erysipelotrichia* origin, *Erysipelotrichia* evolution, *Erysipelotrichia* taxonomy, *Erysipelotrichia* genomics, *Erysipelotrichia* pangenome, *Erysipelotrichia* metabolism, *Erysipelotrichia* phages, *Mycoplasmatota* evolution, genome reduction

## Abstract

*Bulleidia extructa* strain PP_925, isolated from the periodontal pocket of a patient with periodontitis, is a Gram-positive *Bacillota* with an unusually compact genome of 1.38 Mb. Phylogenomic analyses place PP_925 within *Erysipelotrichales* and show close relatedness of *Bulleidia* to *Solobacterium* and *Lactimicrobium,* as well as the existence of previously undescribed related clades. The metabolic repertoire of PP_925 is strongly reduced: it retains glycolysis, the phosphotransacetylase–acetate kinase pathway, and arginine catabolism but lacks the tricarboxylic acid cycle and most de novo biosynthetic pathways for amino acids, nucleotides, fatty acids, cofactors, and vitamins, implying reliance on salvage and cross-feeding. Phylogenetic inference indicates independent peptidoglycan losses in multiple mycoplasma *Erysipelotrichia*-related lineages, while PP_925 has retained an ancestral Gram-positive cell wall despite extensive genomic reduction. The genome preserves systems crucial for host interaction and adaptability, including a horizontally acquired *tad* locus encoding type IV pili, a *comG* competence system, and several adherence-associated virulence factors. Defense mechanisms are diverse and include a CRISPR-Cas II-A system, a type II restriction–modification module adjacent to Gao_Qat-like genes, and the Wadjet system in a genome without prophages; CRISPR spacers indicate repeated encounters with *Bacillota* phages. Comparative genomics of PP_925 and related strains reveals a small core genome with lineage-specific adhesion and defense modules, indicating recent shared ancestry combined with adaptive flexibility under substantial genome reduction.

## 1. Introduction

Progress in culturing methods over recent decades has significantly advanced the isolation of fastidious, strictly anaerobic bacteria from the human microbiota [1,2]. This has led to a substantial increase in the number of cultured and phenotypically characterized bacterial taxa that were previously considered ‘unculturable’. In the human oral and gut microbiota, particularly among adults, the highest biodiversity is observed within the phylum *Bacillota*, primarily represented by the families *Lachnospiraceae*, *Oscillospiraceae*, and *Erysipelotrichaceae* of the class *Clostridia*. Numerous studies are currently aimed at elucidating the potential beneficial roles of species from these families in host health [3,4]. These roles include their contribution to controlling the host’s energy balance and immune regulation. Furthermore, various genera and species within these families have been associated with several human diseases, including metabolic syndrome, obesity, diabetes, liver diseases, inflammatory bowel syndrome, and oral inflammatory diseases such as periodontitis and peri-implantitis [5,6]. Research into the potential of microbiome-targeted therapy is increasingly incorporating multi-omics approaches to study these slow-growing, low-GC, Gram-positive organisms. These studies are revealing their significant ecological versatility [7,8].

Among the various bacterial lineages within *Bacillota*, *Erysipelotrichia* remains one of the most understudied. Phylogenomic studies place *Erysipelotrichia* in a clade that also includes *Mycoplasma* and other wall-less organisms that branch from within the lineage [9,10]. Yet all characterized *Erysipelotrichia* retain a peptidoglycan cell wall [9,10,11,12,13,14,15,16,17], and even their smallest chromosomes, e.g., ≈1.4 Mbp in *B. extructa*, are substantially larger than the ≈0.5 Mbp genomes of the tiniest *Mycoplasmatales.*

Genome reduction is a widespread evolutionary strategy in bacteria, emerging under different ecological pressures. In free-living marine bacteria, particularly those in nutrient-poor ecosystems, selection favors compact genomes with reduced non-essential DNA. This process, known as genome streamlining, enhances replication efficiency in large populations and is well documented in models like *Prochlorococcus* and *Pelagibacter ubique* [18]. On the other hand, host-associated bacteria (e.g., symbionts, commensals) often lose key anabolic functions and rely on salvage mechanisms, driven by small population sizes and relaxed selection, as reviewed in studies of insect symbionts and other intracellular taxa [19,20]. Within the *Bacillota*, the class *Erysipelotrichia* exhibits a remarkable spectrum of genome sizes and metabolic capabilities. While some members maintain versatile genomes, others show significant reductions; yet, unlike *Mycoplasmatota*, they typically retain a Gram-positive cell wall. Phylogenomic evidence indicates *Mycoplasmatota* are polyphyletic, derived independently from *Erysipelotrichia*-like ancestors, suggesting multiple parallel instances of genome reduction in this lineage [9,21].

*Bulleidia extructa* is currently the sole validated species of the genus *Bulleidia*, the type strain of *B. extructa* is represented by strain W1219^T^ [22]. A Gram-positive, non-spore-forming obligate anaerobe, it was first isolated from advanced periodontitis pockets and assigned to the family *Erysipelotrichaceae* as a lineage distinct from *Erysipelothrix* and *Holdemania* [22]. 16S-rRNA surveys reveal *B. extructa* as a rare yet persistent member of the human oral microbiome, enriched in deep periodontal pockets and smoker-associated plaque, while shotgun metatranscriptomics associates its transcripts to metabolically active biofilms in periodontitis lesions [23]. Outside the oral cavity, the species appears, typically at low abundance, in faecal, duodenal, and vaginal microbiomes, suggesting broader anaerobic versatility [11]. Several studies report elevated relative abundance in cohorts with inflammatory bowel disease or schizophrenia, hinting at a possible gut–brain axis connection that warrants systematic investigation [24].

Although isolation in pure culture is uncommon, *B. extructa* has been recovered from dento-alveolar abscesses, brain abscess material, and even a prosthetic hip infection. In the latter case, the strain proved uniformly susceptible to penicillin, clindamycin, and metronidazole, and clinical resolution followed hardware removal plus targeted therapy [25]. These scattered but telling observations, together with their frequent co-occurrence alongside *Porphyromonas* or *Fusobacterium*, point to an opportunistic rather than obligate pathogenesis: the bacterium seldom acts alone, yet when it joins a polymicrobial consortium, it can amplify tissue damage.

Robust conclusions about its pathogenic potential are hampered by the absence of controlled virulence assays, population-scale prevalence studies, and experimental validation of metabolic predictions. Closing these gaps will clarify to what extent *B. extructa* behaves as a low-abundance commensal hitchhiker within subgingival biofilms and/or as a slow-growing opportunistic contributor to chronic anaerobic infections. Comprehensive analysis, including in silico, in vitro, and in vivo approaches, is therefore required to elucidate its lifestyle, pathogenicity, and ecological roles.

An important aspect of understanding the lifestyle and potential pathogenicity of bacteria with reduced genomes is the presence of virulence-associated loci that mediate host interaction. Among these, type IV filament systems, such as the *tad*, are widely distributed in bacterial and archaeal genomes and frequently acquired via horizontal gene transfer [26]. *Tad* pili play critical roles in adherence, colonization, and biofilm formation in diverse organisms [26,27,28,29,30]. Even in highly reduced host-dependent lineages, type IV pili are often retained and serve essential functions in attachment and colonization [31,32,33,34]. In Gram-positive bacteria, type IV pili and competence-associated filament systems facilitate adhesion, motility, and DNA uptake, contributing to both colonization and horizontal gene transfer [32,33,34].

In this study, we describe the isolation, cultivation, and genomic characterization of *Bulleidia extructa* strain PP_925, a rare anaerobic, cell wall-containing bacterium with one of the smallest genomes in its class (approximately 1.4 Mb). Through phylogenetic, pangenome, and metabolic analyses, we uncover evidence of multiple genome reduction events within *Erysipelotrichales* and *Mycoplasmatota*, with different gene-loss strategies. We demonstrate that *B. extructa* retains a *tad* locus (likely horizontally acquired) and a competence-associated locus, along with multiple adhesion-related virulence factors and anti-phage defense systems, despite lacking intact prophages. CRISPR analysis reveals exposure to groups of phages infecting *Bacillota*. Metabolically, the organism retains only glycolysis, acetate fermentation (Pta-AckA), and arginine degradation, but has lost most biosynthetic pathways, instead relying on salvage. Together, these findings position *B. extructa* as a compelling model for minimalist adaptation in Gram-positive bacteria, highlighting parallel reductive paths to *Mycoplasmatota* and offering insights into host-associated microbial evolution.

## 2. Results

### 2.1. Phenotypic Features

**Figure 1:**
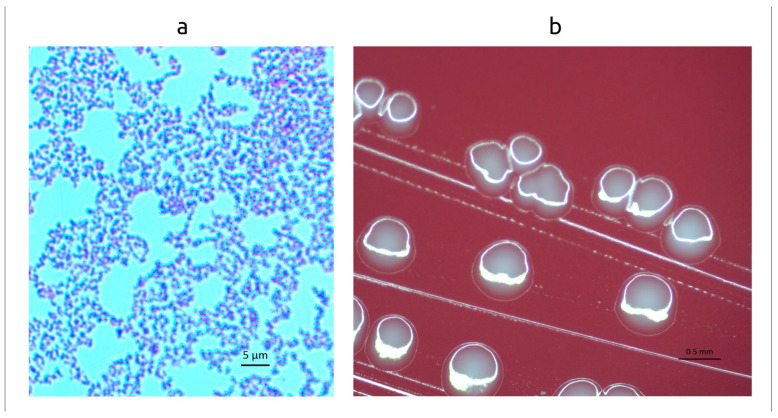
Morphology of *Bulleidia extructa* PP_925: (**a**) Gram stain, 96 h of incubation. The scale bar is 5 µm. (**b**) Round, white, and shiny colonies on Anaerobe basal agar (Oxoid) plates, after 6 days of incubation. The scale bar is 0.5 mm.

Cells of strain PP_925 were obligately anaerobic, non-spore-forming, non-motile, Gram-positive, coccobacillus rods. Colonies on Anaerobe Basal Agar (Oxoid) plates after six days of incubation at 37 °C under anaerobic conditions were 0.6–0.8 mm in diameter, round, white, shiny, and flattened with an entire margin. In areas of dense growth, there was weak beta-hemolysis on the media with defibrinated sheep blood. In colonies from 96 h plates, Gram-stained cells are typically 0.4–0.45  ×  0.5–0.6 μm in size and occur in pairs or clusters (Figure 1).

**Figure 2:**
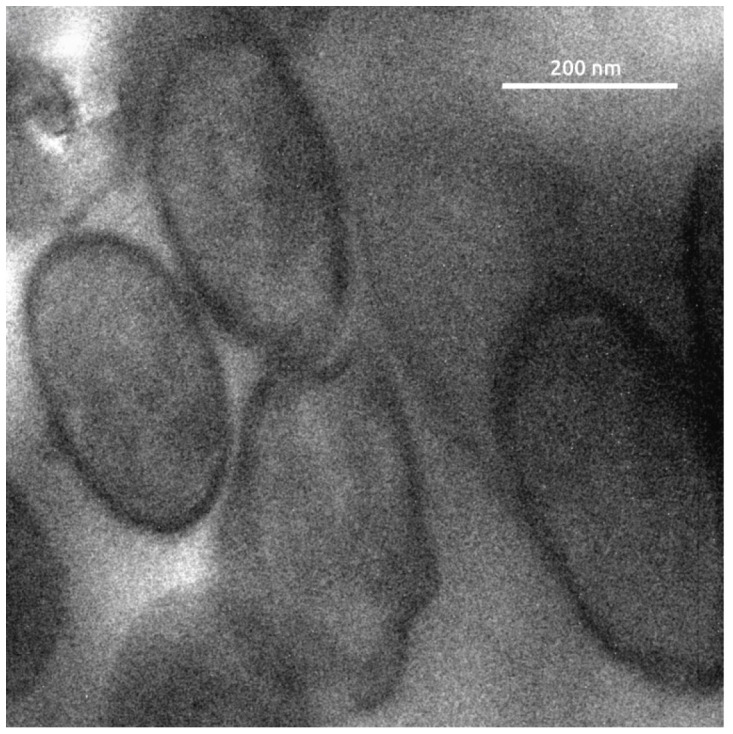
Transmission electron microscopy of bacteria grown in liquid medium after 72 h of incubation. The scale bar is 200 nm.

Transmission electron micrographs (Figure 2) of ultrathin sections of the cells revealed a typical Gram-positive monoderm cell envelope consisting of a cytoplasmic membrane and a peptidoglycan layer, and also demonstrated the absence of endospore formation.

From the API 20A test results, *Bulleidia extructa* PP_925 did not produce acid from any of the tested carbohydrates, including D-lactose, D-sucrose, D-maltose, D-xylose, L-arabinose, D-cellobiose, D-melezitose, D-raffinose, L-rhamnose, D-trehalose, D-glucose, D-mannitol, salicin, glycerol, D-mannose, and D-sorbitol. Additionally, tests for urease and indole production were negaative.

In the Rapid ID 32A identification panel, based on the use of chromogenic enzyme substrates, strain PP_925 demonstrated negative reactions for glycoside hydrolases, including α-galactosidase, β-galactosidase, β-galactosidase 6-phosphate, α-glucosidase, β-glucosidase, α-arabinosidase, β-glucuronidase, N-acetyl-β-glucosaminidase, and α-fucosidase. Carbohydrate fermentation reactions for mannose and raffinose, included in this panel, yielded negative results. Additionally, the strain demonstrated positive reactions for a number of arylamidases (e.g., arginine arylamidase, leucine arylamidase, histidine arylamidase, and tyrosine arylamidase (weak)). Alkaline phosphatase tested negative. Pyroglutamic acid arylamidase, nitrate reductase, arginine dihydrolase, and glutamate decarboxylase reactions were negative.

In disc-diffusion experiments, strain PP_925 was resistant to gentamicin and amikacin but sensitive to penicillin G, amoxicillin/clavulanic acid, ampicillin/sulbactam, ceftazidime, clindamycin, doxycycline hydrochloride, azithromycin, levofloxacin, metronidazole, and vancomycin (Supplementary Table S1).

### 2.2. Phylogeny and Taxonomy

#### 2.2.1. Overview of Sequencing, Assembly, and Annotation for B. extructa PP_925

Whole-genome sequencing of *Bulleidia extructa* PP_925 resulted in an assembly of 20 contigs with a total sequence length of 1,377,588 bp (Contig N50, 217,216 bp) and 378.0 x coverage (NCBI GenBank accession number JBMPNY000000000.1). The GC content of the assembly was 36.1%. Annotation predicted 1335 protein-coding genes (coding sequences, CDSs); 41 tRNA genes; 1 tmRNA gene; and 3 genes corresponding to full-length 16S, 23S, and 5S rRNAs. In addition, one 3333 bp repeat region and two sequences corresponding to ncRNAs (RNase P RNA component class B and the small-type signal recognition particle sRNA) were identified in the genome. Functional assignment suggested putative functions or protein family affiliations for 1111 encoded proteins, while 224 genes were annotated as hypothetical protein CDSs. The coding density was 90.7%. Assembly completeness was estimated at 98.67%, and the predicted contamination level was 0.67%.

#### 2.2.2. 16S rRNA Gene Sequence Analysis and Phylogeny

At present, only a single validly published species, *Bulleidia extructa*, is recognized within the genus, represented by the type strain W1219^T^ (https://bacdive.dsmz.de/strain/5365, accessed 15 September 2025) [22]. However, public sequence repositories contain several additional 16S rRNA gene sequences and genomic assemblies annotated as “Bulleidia”, many of them originating from culture-independent microbiome surveys, and have not yet been formally validated at the species level. To place strain PP_925 in this broader context, we extracted its 16S rRNA gene from the genome and used it as a query in BLAST+ 2.12.0 searches against the NCBI GenBank core nucleotide database, and then reconstructed phylogenies that also incorporated related sequences. These searches retrieved multiple homologous *Bulleidia* sequences (Supplementary Table S2), which were annotated as *Solobacterium* spp. and *Lactimicrobium massiliense*.

**Figure 3:**
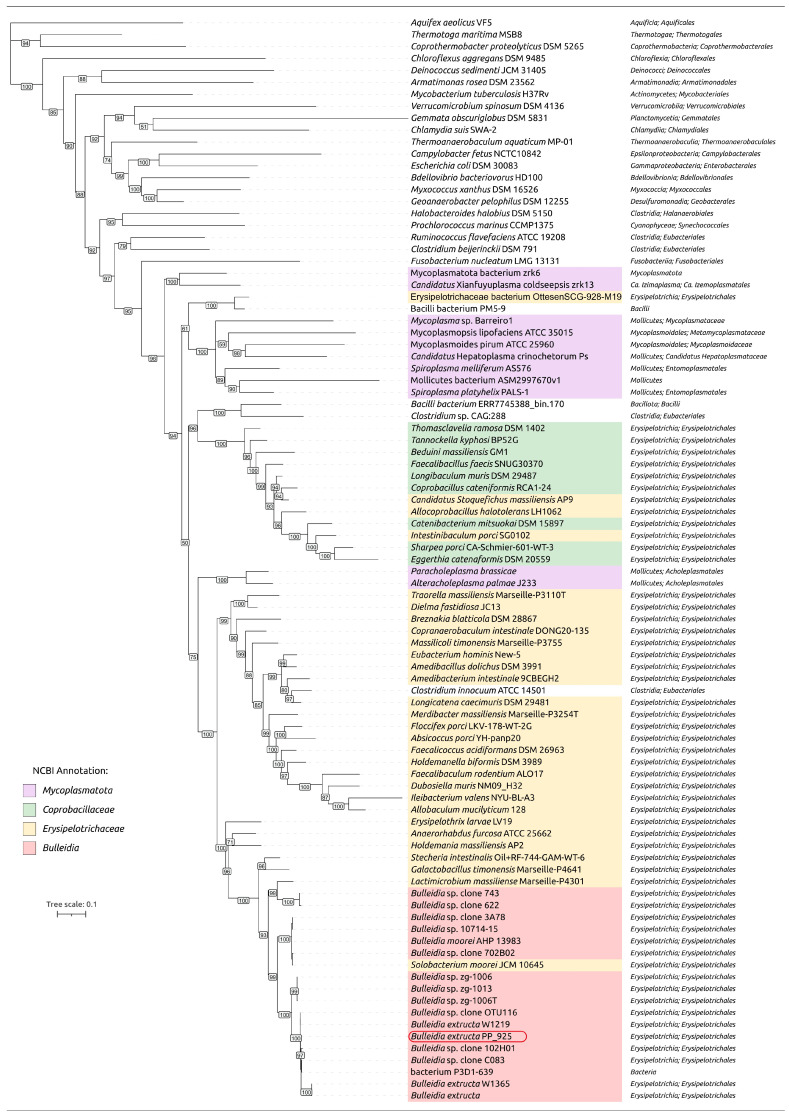
16S rDNA maximum-likelihood phylogenetic tree of *Erysipelotrichales* and related bacteria. The analysis encompasses 100 representative genomes. Branches with bootstrap support < 50% were deleted. Bootstrap values ≥ 50% are shown at nodes. Scale bar: 0.1 substitutions per site. The tree is rooted using *Aquifex aeolicus* VF5, *Thermotoga maritima* MSB8, and *Coprothermobacter proteolyticus* DSM 5265 as outgroups. NCBI taxonomy labels are attached to bacterial names, with group coding in the legend. The strain *B. extructa* PP_925 is outlined in red. GenBank accession numbers for all sequences included in the tree are listed in Supplementary Table S3.

Phylogenetic analysis was carried out with these sequences and representatives of different bacterial taxa (Supplementary Table S3), including members of the class *Erysipelotrichia* (Figure 3). The analysis placed *B. extructa* PP_925 together with seven sequences showing 98.8–99.9% identity to it in a branch that was sister to another cluster containing three sequences annotated as *Bulleidia* spp., with identities of 97.1–97.3%. This combined clade was adjacent to a branch that included *Solobacterium moorei* JCM 10645^T^, the type strain of the officially recognized species *S. moorei* [35]. Sequences within this clade showed 93.3–93.8% identity to *B. extructa* PP_925.

Two further sequences, annotated as “Uncultured *Bulleidia*” spp. but sharing only 90.4% pairwise identity with *B. extructa* PP_925, clustered instead with *Lactimicrobium massiliense*. In this phylogenetic reconstruction, *Solobacterium moorei* and its closest relatives appear more closely related to *Bulleidia* than to other genera of *Erysipelotrichia*. Applying the frequently used thresholds of 98.5% for species-level similarity [36] and 94.5% for genus delineation [37], the available 16S rRNA gene sequences suggest the presence of three putative species-level lineages within the genus *Bulleidia* together with *Solobacterium moorei*. However, several sequences are partial, and the pairwise identities of the inferred new *Bulleidia* lineages to *Bulleidia extructa* W1219^T^ are close to these thresholds; therefore, any species-level assignments should be regarded as preliminary and will require more detailed analyses, including genome-wide similarity comparisons, to be substantiated (Supplementary Table S2). Notably, the analysis also highlights the polyphyly of *Mycoplasmatota*, a point of considerable interest for evolutionary discussions [38,39].

#### 2.2.3. GTDB-Tk Analysis

A representative dataset for phylogenetic analyses based on signature proteins was compiled using the Genome Taxonomy Database (GTDB) and the GTDB-Tk taxonomic classification pipeline. Preliminary phylogenetic analysis of *Bacillota* bacteria was carried out with concatenated alignments generated by GTDB-Tk, followed by tree reconstruction using FastTree. The dataset included 4431 sequences, of which 371 were annotated by GTDB as members of the family *Erysipelotrichaceae* and 66 as members of the family *Coprobacillaceae* (both within the order *Erysipelotrichales*), giving a total of 437 sequences. These two families formed sister monophyletic branches that together constituted a clade adjacent to another clade comprising four unclassified bacterial genomes: Pq454_bin41 (genome size 1.2 Mbp), Md513_bin19 (1.3 Mbp), OttesenSCG-928-M19 (1.1 Mbp), and PM5–9 (1.6 Mbp).

These four unclassified bacteria, hereafter referred to as “Clade A,” together with their closest relative in the GTDB-Tk tree, *Bacilli* bacterium isolate HGM10900 assigned by GTDB-Tk to the genus *Candidatus* Caccosoma (recently proposed in [40]), formed the final dataset of 442 strains (Supplementary Table S4). This relative was designated as the outgroup in subsequent analyses.

**Figure 4:**
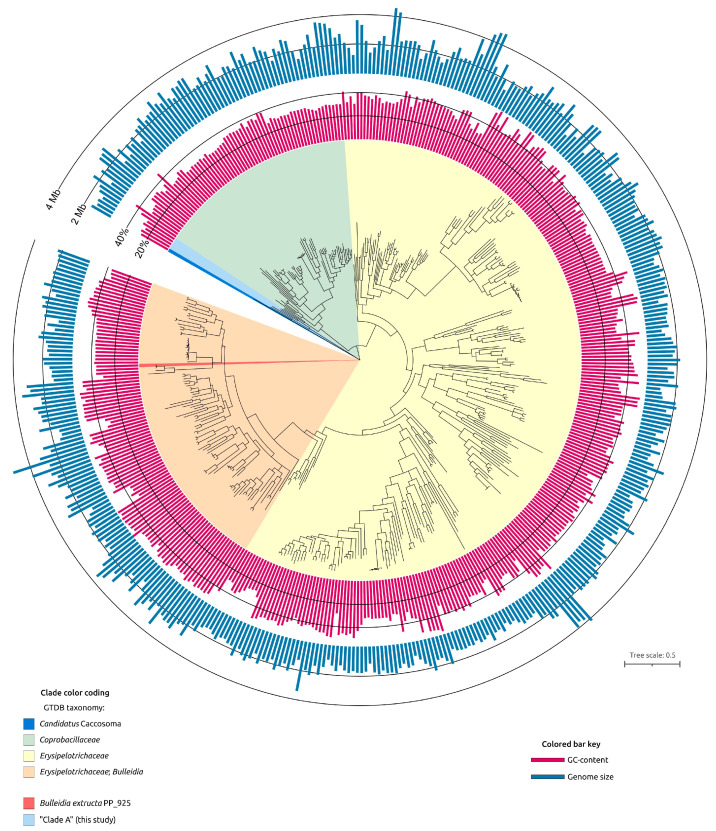
Maximum-likelihood phylogenetic tree of *Erysipelotrichales* and related bacteria reconstructed using GTDB alignments of the BAC120 conserved protein marker set. The analysis included 442 representative genomes described in the text. Genome size is represented by blue bars with a scale shown next to each lane, and GC content is represented by purple bars with a separate scale. Branches with bootstrap support values below 50% were collapsed. Bootstrap values of 50% or higher are shown at the corresponding nodes. The scale bar corresponds to 0.2 substitutions per site. The tree is rooted with the *Bacilli* bacterium isolate HGM10900 as the outgroup. The composition of Clade A is described in the text. GTDB taxonomy is indicated in the legend. GenBank accession numbers for all sequences included in the tree are listed in Supplementary Table S4.

GTDB-Tk phylogenetic analysis indicated the placement of *B. extructa* PP_925 within the family *Erysipelotrichaceae* and its close relatedness to the type strain *Bulleidia extructa* W1219^T^ (Figure 4). However, validly published classifications and NCBI taxonomy annotations differ from the GTDB-based taxonomy. For example, GTDB assigns several groups of bacteria that are usually considered distinct genera to the genus *Bulleidia*. These include *Solobacterium moorei* [41], annotated as “*Bulleidia moorei*”; *Lactimicrobium massiliense* [42], annotated as “*Bulleidia massiliense*”; *Galactobacillus timonensis* [42], annotated as “*Bulleidia timonensis*”; and *Stecheria intestinalis* [43], annotated as “*Bulleidia intestinalis*”.

General characteristics of the 437 genomes assigned by GTDB to the order *Erysipelotrichales* are highly variable. Genome sizes range from 916 kbp to 5.208 Mbp, with a median of 2.003 Mbp, and GC content ranges from 27.0% to 56.5%, with a median of 38.6%. Overall, the genome of *Bulleidia extructa* PP_925 (1.377 Mbp) is relatively small compared to most *Erysipelotrichales* genomes, while its GC content is typical for the order.

**Figure 5:**
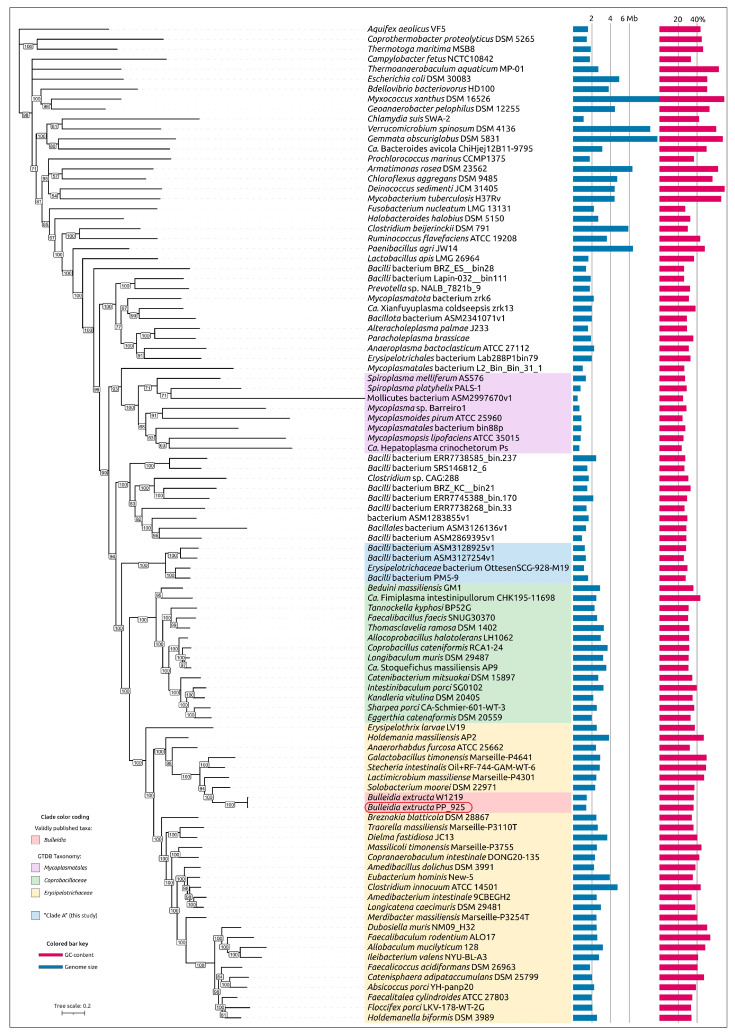
Maximum-likelihood phylogenetic tree of *Erysipelotrichales* and related bacteria reconstructed using GTDB alignments of the BAC120 conserved protein marker set. The analysis included 100 representative genomes. Genome size is represented by blue bars with a scale shown above the bars, and GC content is represented by purple bars with a separate scale above the bars. Branches with bootstrap support values below 50% were collapsed. Bootstrap values of 50% or higher are shown at the nodes. The scale bar represents 0.2 substitutions per site. The tree was rooted with *Aquifex aeolicus* VF5, *Thermotoga maritima* MSB8, and *Coprothermobacter proteolyticus* DSM 5265 as outgroups. The composition of Clade A is described in the text. The strain *B. extructa* PP_925 is outlined in red. GTDB taxonomy is indicated in the legend. GenBank accession numbers for all sequences included in the tree are listed in Supplementary Table S3.

To show the phylogenetic position of *Bulleidia extructa* PP_925 and to resolve relationships at both higher and lower taxonomic ranks, a set of 100 genomes (Supplementary Table S3) was analyzed using the GTDB alignments of the BAC120 conserved protein marker set. This expanded dataset included representatives of higher bacterial taxa, such as *Mycoplasmatota*, which are evolutionarily related to the class *Erysipelotrichia*, and several genera within the family *Erysipelotrichaceae*. The analysis (Figure 5) indicated the closest affinity of *Bulleidia* to *Solobacterium* and to a lesser extent to *Lactimicrobium*, *Galactobacillus,* and *Stecheria*, in agreement with the 16S rRNA phylogeny (Figure 3). Both the GTDB phylogenies and the 16S rRNA phylogeny highlight the polyphyletic character of *Mycoplasmatota* and their close evolutionary relationship to *Erysipelotrichia*. Notably, the average genome size of bacteria within this clade, assigned by GTDB to *Mycoplasmatota*, is more than two times smaller than the average genome size of GTDB-classified *Mycoplasmatota* from the other clades, which also contain non-*Mycoplasmatota* bacteria.

#### 2.2.4. Ribosomal Proteins Phylogeny. GTDB and NCBI Taxonomic Assignments

**Figure 6:**
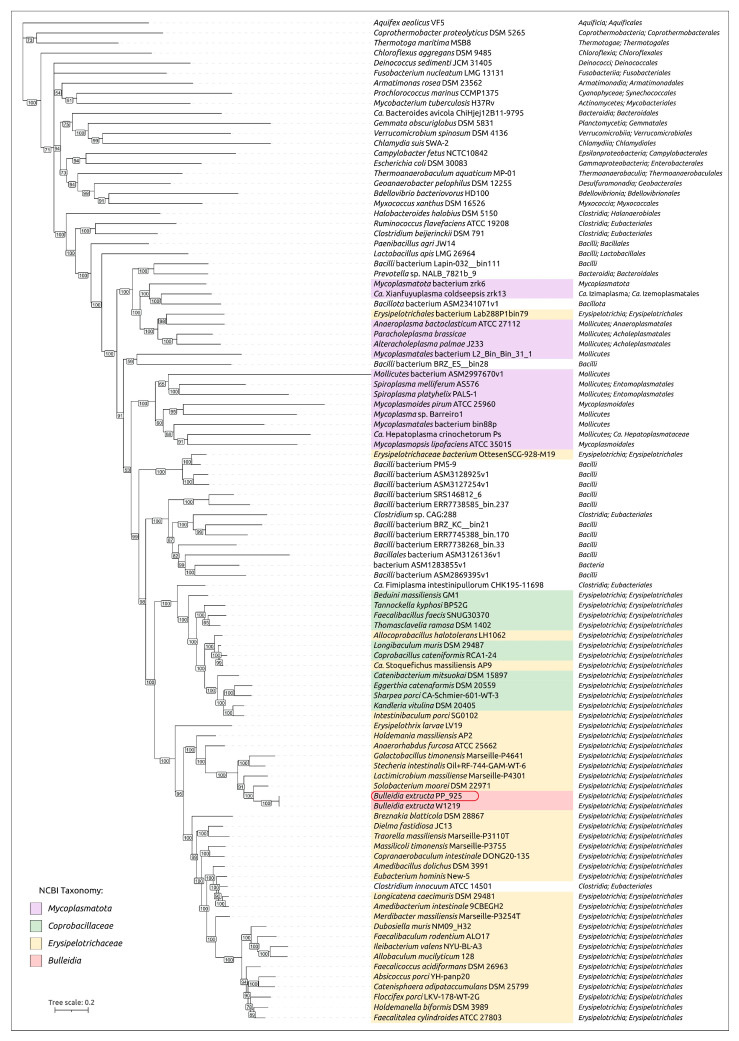
Maximum-likelihood phylogenetic tree of *Erysipelotrichales* and related bacteria reconstructed using concatenated alignments of ribosomal proteins. The analysis included 100 representative genomes. Branches with bootstrap support values below 50% were collapsed. Bootstrap values of 50% or higher are shown at the nodes. The scale bar corresponds to 0.2 substitutions per site. The tree was rooted with *Aquifex aeolicus* VF5, *Thermotoga maritima* MSB8, and *Coprothermobacter proteolyticus* DSM 5265 as outgroups. NCBI taxonomy labels are displayed next to bacterial names, and group coding is shown in the legend. The strain *B. extructa* PP_925 is outlined in red. GenBank accession numbers for all sequences included in the tree are listed in Supplementary Table S3.

Phylogenetic analysis based on concatenated alignments of 42 ribosomal proteins from the same genomes used in the previously discussed GTDB tree revealed a topology similar to the GTDB-based reconstruction (Figure 6). The tree indicated the relatedness of *Bulleidia* to representatives of *Solobacterium*, *Lactimicrobium*, *Galactobacillus,* and *Stecheria*. It also showed that *Mycoplasmatota* are non-monophyletic and closely related to *Erysipelotrichia*. Importantly, taxonomic assignments obtained from NCBI taxonomy and GTDB are conflicting in several cases. The phylogenetic analysis, however, confirmed the consistency of tree topology in terms of the monophyly of corresponding branches according to the GTDB classification, while highlighting discrepancies between this topology and several NCBI taxonomy annotations.

#### 2.2.5. Average Nucleotide Identity (ANI)

**Figure 7:**
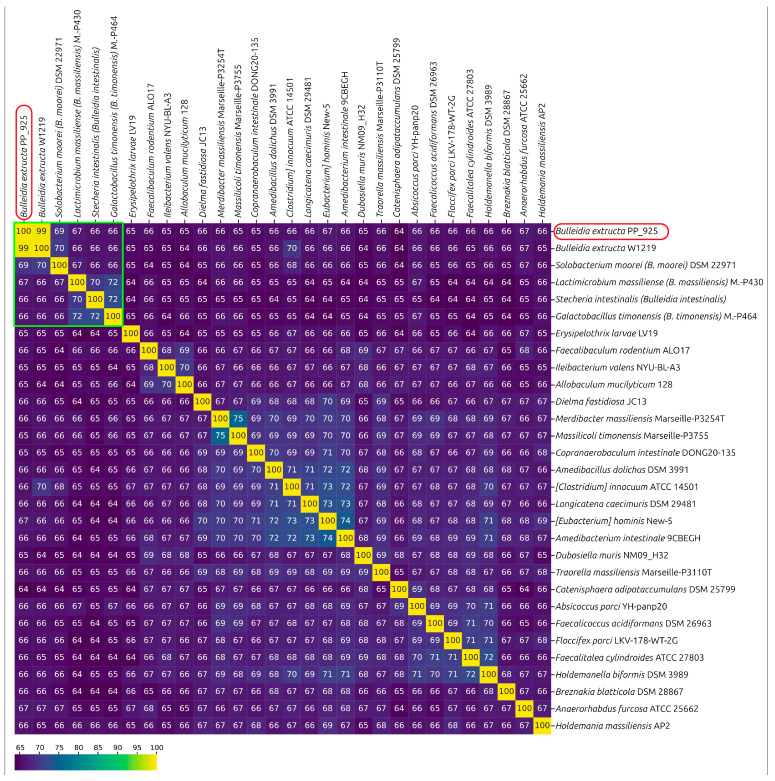
Clustered heatmaps of ANI across 30 bacterial genomes, featuring *Bulleidia extructa* PP_925 and related *Erysipelotrichaceae*, group classified by the GTDB as “*Bulleidia*” outlined in green. The name of strain *B. extructa* PP_925 is outlined in red. Expanded heatmaps and numerical matrix (larger dataset): Supplementary Figure S1 and Table S5.

Calculations of ANI confirmed the close relatedness of *Bulleidia extructa* PP_925 to *Bulleidia extructa* W1219^T^, with an ANI value of 99.1% (Supplementary Table S5), which is significantly higher than the accepted species delineation threshold of 95–96% based on genomic data [36,44]. No other analyzed genomes reached a level of similarity sufficient to classify them within the *B. extructa* species. The next highest ANI values were observed for the genome labeled as “*Bulleidia* sp. zg-1006” (GCF_016812035, ANI 80.2%) and for a metagenomic assembly annotated as “uncultured *Bulleidia* sp. genome assembly SRR11749279_bin.12_metaWRAP_v1.3_MAG” (GCA_947087645, ANI 72.9%). All other analyzed sequences had ANI values below 70% (Figure 7).

#### 2.2.6. Average Amino Acid Identity (AAI) and Percentage of Conserved Proteins (POCP)

As proposed earlier [45,46], AAI and POCP calculations were conducted to evaluate genus-level delineation. These metrics are generally correlated [47]. For AAI, genus delineation thresholds ranging from 58% to 65% have been suggested in different studies [47,48,49], while for POCP, a 50% threshold has been proposed [46]. However, the appropriateness of the 50% POCP threshold, as well as the applicability of this metric as the primary criterion, has been questioned [47].

**Figure 8:**
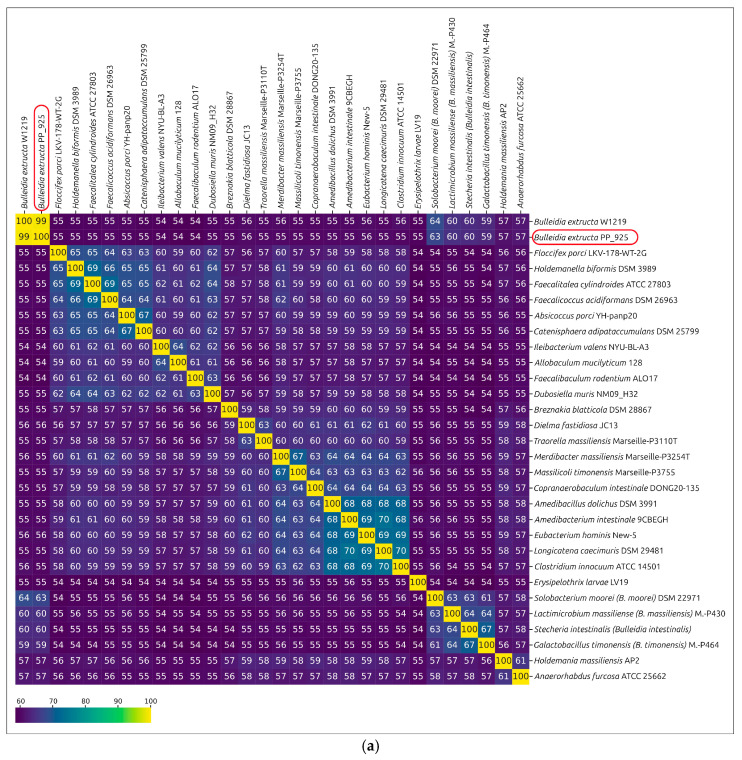

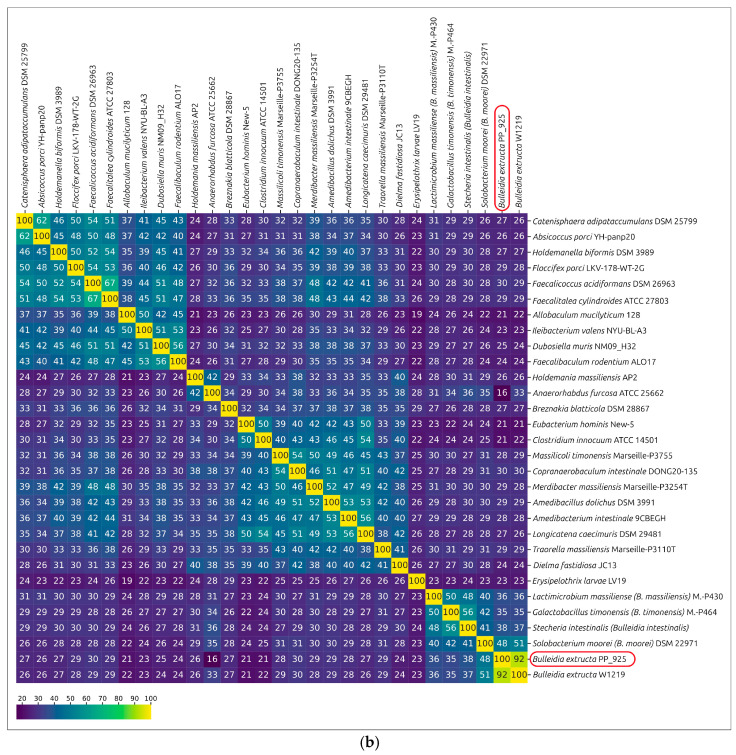
Clustered heatmaps of genomic similarity metrics across 30 bacterial genomes, featuring *Bulleidia extructa* PP_925 and related *Erysipelotrichaceae*: (**a**) AAI values. (**b**) POCP values. The name of strain *B. extructa* PP_925 is outlined in red. Expanded heatmaps and numerical matrices (larger dataset): Supplementary Figures S2 and S3, Supplementary Tables S6 and S7.

*Bulleidia extructa* W1219^T^ and *Solobacterium moorei* DSM 2297^T^ show an AAI of nearly 64.5%, and approximately 60% when compared to *Lactimicrobium massiliense*, *Galactobacillus timonensis,* and *Stecheria intestinalis* (Figure 8a). Four genomes, including *B. extructa* PP_925, *Bulleidia* sp. zg-1006, *B. extructa* W1219^T^, and the metagenome-assembled genome annotated as “*Bulleidia* sp.” (GCA_947087645), formed a distinct cluster with AAI values above 70% (Supplementary Table S6). In a similar way, these four genomes also grouped together in a separate cluster with POCP values above 63%.

These four bacteria, together with *Solobacterium moorei*, *Lactimicrobium massiliense*, *Galactobacillus timonensis,* and *Stecheria intestinalis*, are members of a larger cluster, with POCP values between them apparently higher than with other analyzed genomes (Figure 8b, Supplementary Table S6).

The results of ANI, AAI, and POCP analyses are consistent with the phylogenetic reconstructions and support the delineation of the four analyzed strains (*Bulleidia extructa* PP_925, *Bulleidia* sp. zg-1006, *B. extructa* W1219^T^, and the metagenome-assembled genome annotated as “*Bulleidia* sp.” GCA_947087645) within the genus *Bulleidia*, based on the AAI and POCP values that are applied for the delineation of the closely related genera *Solobacterium*, *Lactimicrobium*, *Galactobacillus*, and *Stecheria*. Furthermore, based on the genomic data and ANI calculations, three *Bulleidia* species can be delineated. The first corresponds to the already classified *B. extructa* PP_925 and *B. extructa* W1219^T^, the second is represented by the isolated strain *Bulleidia* sp. zg-1006, and the third is represented by the metagenome-assembled genome “uncultured *Bulleidia* sp. genome assembly SRR11749279_bin.12_metaWRAP_v1.3_MAG” (GCA_947087645).

### 2.3. Common and Characteristic Gene Clusters

The genome of *Bulleidia extructa* PP_925 was analyzed for the presence of gene clusters associated with cellular processes that are important for the general characterization of the bacterium and related groups, as well as for distinguishing *B. extructa* PP_925 and closely related bacteria from other taxa. The genome was also examined for genes associated with antibiotic and phage resistance, which are of therapeutic relevance. Special attention was given to the relationships between the genes and proteins of *B. extructa* PP_925 and those of other organisms.

#### 2.3.1. Cell Wall Biosynthesis

To the best of current knowledge, the family *Erysipelotrichaceae* includes Gram-positive bacteria with a cell envelope consisting of a cytoplasmic membrane covered by a distinct layer of peptidoglycan (PG) cell wall. This structure is clearly visible in the earlier published TEM images of *Bulleidia extructa* and *Solobacterium moorei* [22,50] as well as in the present study. Although cell-wall-less mycoplasmas are phylogenetically close to *Erysipelotrichia*, no cell-wall-less representatives of *Erysipelotrichia* are known at present. The absence of peptidoglycan closer to the root of phylogenetic trees raises questions about the evolutionary origin of these genes in *Erysipelotrichia*.

To investigate the evolutionary history of peptidoglycan biosynthesis in *B. extructa* PP_925, twelve enzymes known to participate in the stepwise synthesis of the UDP-MurNAc-pentapeptide precursor or peptidoglycan assembly were analyzed [51,52,53] (Table 1).

**Figure 9:**
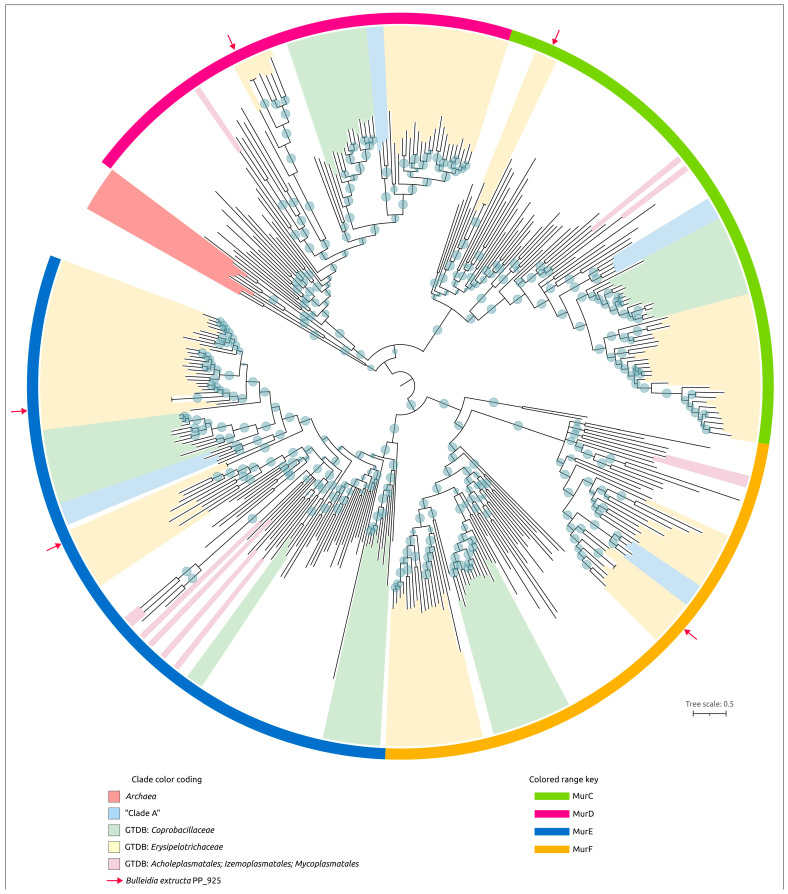
Maximum-likelihood phylogenetic tree constructed using alignments of amino acid sequences of MurC, MurD, MurE, and MurF proteins homologous to the corresponding *Bulleidia extructa* PP_925 proteins identified in 100 representative genomes of *Erysipelotrichia*, other bacteria, and cultivated archaea. Branches with bootstrap support values below 50% were collapsed. Bootstrap support values of 50% or higher are shown at the nodes, with circles marking the corresponding branches. Positions of *Bulleidia extructa* PP_925 are indicated with red arrows outside the circle. The scale bar represents 0.5 substitutions per site. The tree is rooted at the midpoint. The composition of Clade A is described in the text. GTDB taxonomy is indicated in the legend. The same tree with labels including organism names is shown in Supplementary Figure S4.

**Figure 10:**
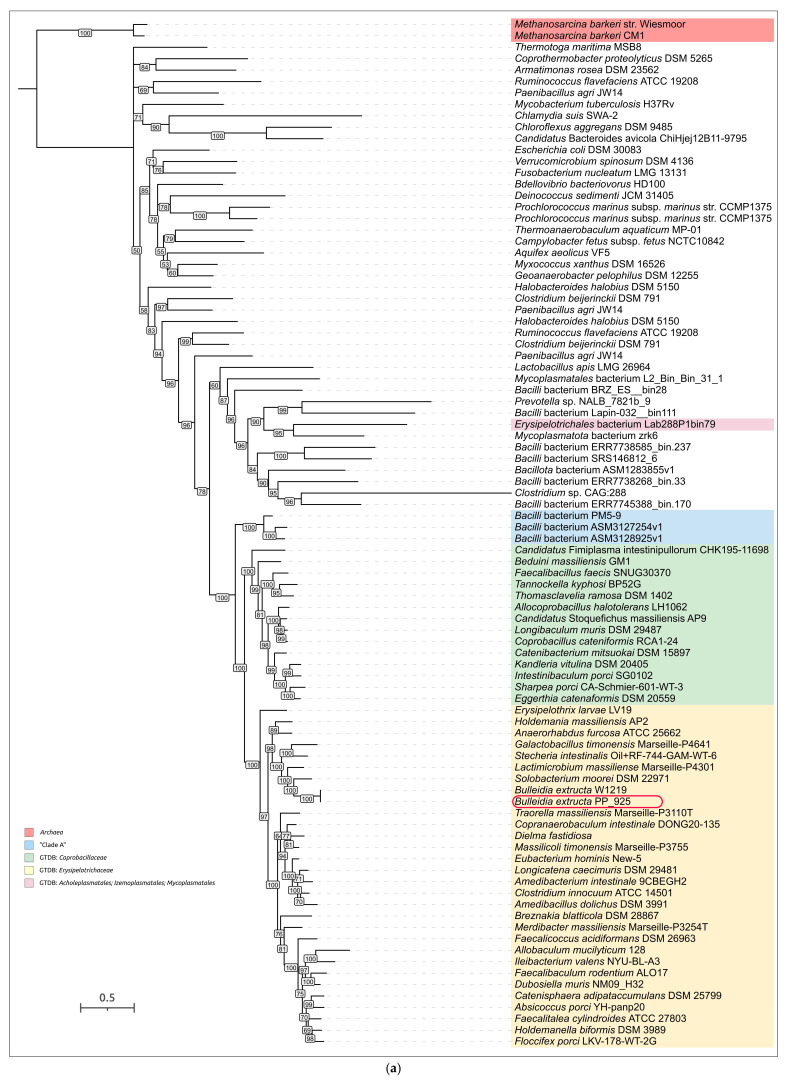

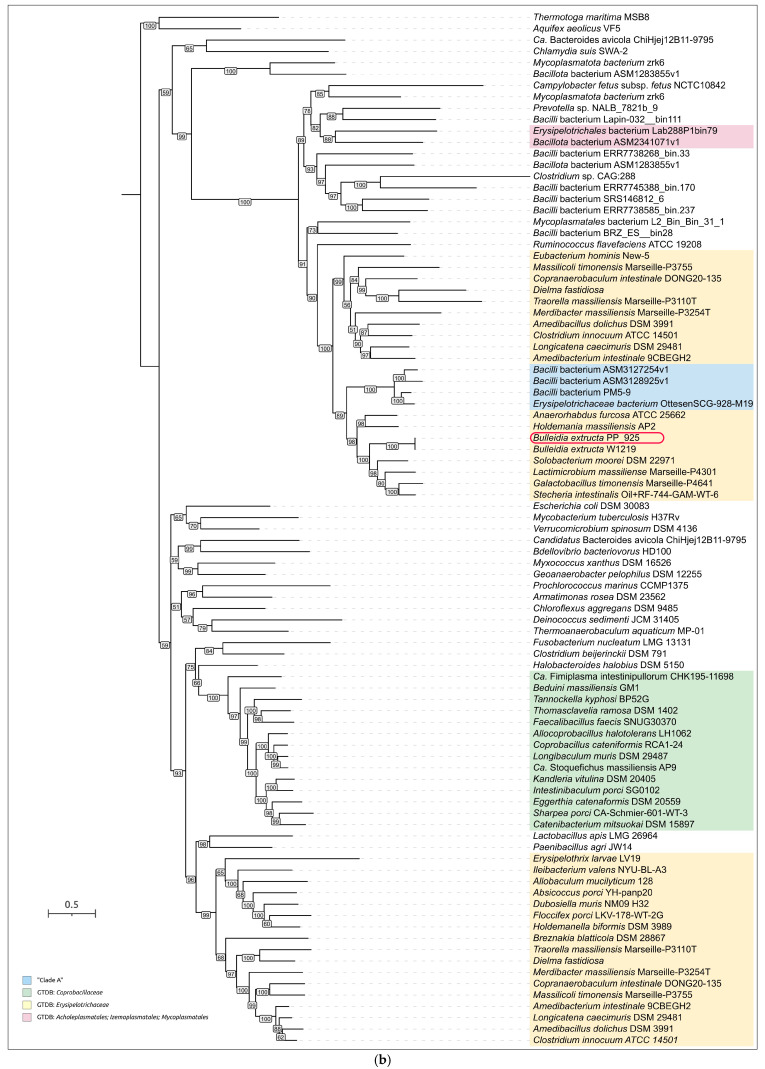

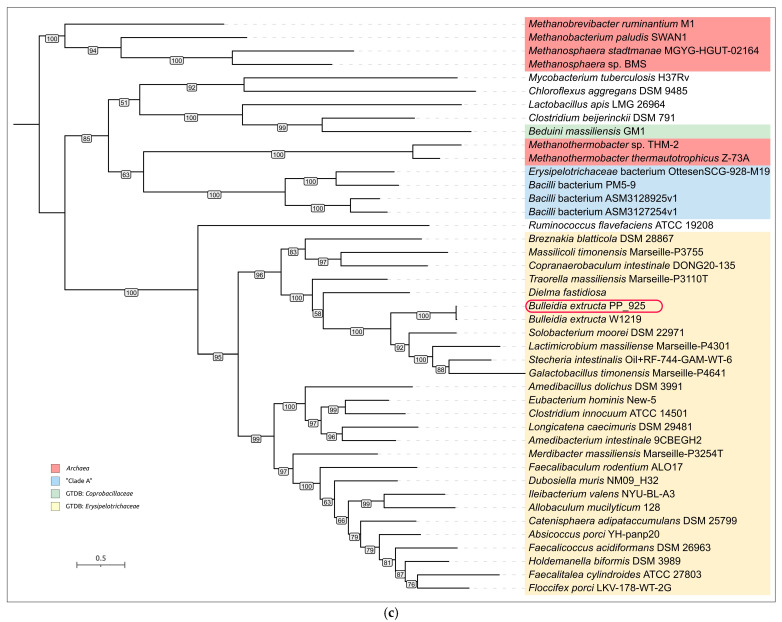
Maximum-likelihood phylogenetic trees of *Erysipelotrichales* and other prokaryotes constructed using alignments of amino acid sequences of MurA, MurF, and MurT proteins homologous to the corresponding *Bulleidia extructa* PP_925 proteins. Branches with bootstrap support values below 50% were collapsed. Bootstrap support values of 50% or higher are shown at the nodes. The scale bar represents 0.5 substitutions per site. The composition of Clade A is described in the text. (**a**) MurA tree, rooted with *Methanosarcina* species. (**b**) MurF tree, rooted with *Thermotoga maritima* and *Aquifex aeolicus*. (**c**) MurT tree, rooted with *Methanobrevibacter* and *Methanosphaera* species. The strain *B. extructa* PP_925 is outlined in red.

Homologs of these proteins encoded in the genome of *B. extructa* PP_925 were searched using BLAST (E-value cut-off 10^−5^) across 100 representative genomes of *Erysipelotrichia, Mycoplasmatota,* and other bacterial taxa used in the previous analyses (Figure 3, Figure 4, Figure 5 and Figure 6). The searches for MurC, MurD, MurE, and MurF yielded partially overlapping results, reflecting their functional similarity and possible evolutionary relatedness. Some annotations conflicted with those of homologous sequences, and therefore a preliminary clustering was performed by constructing a phylogenetic tree that included all four proteins (Figure 9, with organism names removed; the complete tree is shown in Supplementary Figure S4). In addition, several homologs from complete archaeal genomes (non-metagenomic), identified in the NCBI nr/nt database, were included to facilitate rooting of the trees. The resulting trees are presented in Figure 10a,b, as well as in Supplementary Figures S5–S14.

Interestingly, the MurCDEF tree groups homologous archaeal sequences apart from bacterial sequences, apparently reflecting differences in the structure of murein and pseudo-murein stem peptides, and places them close to each other, although they are not monophyletic in this reconstruction. The branch containing MurC and MurD is closer to archaeal sequences than the MurE and MurF branch (Figure 9, Supplementary Figure S4).

Homologs of MurA, MurB, MurT, and MraY were also found in genomic sequences of cultured haloarchaeal species, and several were included in phylogenetic analyses for rooting the trees (Figure 10a,b). Homologs of FemA and MurG were not identified in the genomes of cultured archaea using TBLASTN searches against the NCBI nt database.

The MurT phylogeny shows a complex evolutionary history of this protein, which may involve genetic exchanges between archaea and bacteria (Figure 10c). Comparisons of the GTDB and ribosomal protein phylogenies, on the one hand, and the phylogenies of peptidoglycan biosynthesis-associated genes, on the other hand, indicate multiple events of horizontal transfer and gene duplication.

Genomes of several presumably wall-less mycoplasma-like organisms, assigned by GTDB to the order *Mycoplasmatales*, do not contain complete sets of peptidoglycan-associated genes. Notably, the assembly labeled *Erysipelotrichales bacterium* Lab288P1bin79 (GCA_009784525), assigned by GTDB to the order *Acholeplasmatales*, encodes nearly the complete set of peptidoglycan biosynthesis genes, including a non-homologous alanine racemase compared to that of *B. extructa* PP_925. The genome labeled *Bacillota bacterium* ASM2341071v1 (GCA_023410715) also contains most of the examined peptidoglycan-associated genes, despite its GTDB classification to the order *Izemoplasmatales*. It has been proposed that *Candidatus* Izemoplasma represents an intermediate stage in the reductive evolution from *Bacillota* to *Mycoplasmatota* [54].

In contrast, several genomes assigned by GTDB to taxa outside the recognized wall-less orders *Acholeplasmatales*, *Haloplasmatales* [55], *Candidatus* Izemoplasmatales and *Mycoplasmatales*, but phylogenetically close to them, were not found to encode the homologs of twelve analyzed peptidoglycan biosynthesis proteins. These include *Bacillales* bacterium ASM3126136v1 (GCA_031261365), *Bacilli* bacterium ASM2869395v1 (GCA_028693955) and “uncultured *Bacilli* bacterium BRZ_KC__bin21” (GCA_944332075). This group diverged after the wall-less taxa and before Clade A; *Erysipelotrichia* diverged later, after Clade A (Figure 5 and Figure 6). Interestingly, neighboring genomes appear to retain the peptidoglycan-biosynthesis genes that these bacteria lack. These data may indicate the need to investigate whether a cell wall is present in some currently unclassified, uncultured bacteria that occupy a phylogenetically intermediate position between *Mycoplasmatota* and *Erysipelotrichia*.

It is possible that some of these observations result from erroneous assemblies. CheckM2 completeness analysis of the genomic assemblies indicated possible loss of information about certain genes or contamination in some cases. Nevertheless, the reliability of completeness metrics is not absolute, as illustrated by the genome of *Spiroplasma platyhelix* PALS-1 (GCF_021496725, circular chromosome), which was labeled as complete but yielded a CheckM2 completeness estimate of only 92.94%.

Importantly, the group of wall-less taxa (*Acholeplasmatales*, *Haloplasmatales*, *Candidatus* Izemoplasmatales, and *Mycoplasmatales*) is not monophyletic in either the GTDB tree or the ribosomal protein tree. In both cases, the clades containing wall-less branches are preceded by earlier-diverging lineages that include at least several peptidoglycan-associated genes, which are positioned closer to the root of the entire clade with meaningful statistical support. These include assemblies that are misclassified in the NCBI annotations as “*Prevotella* sp. NALB_7821b_9” (GCA_023665755), “*Mycoplasmatota* bacterium zrk6” (GCA_018394315), and “uncultured *Mycoplasmatales* bacterium L2_Bin_Bin_31_1” (GCA_937936465) (Figure 10).

#### 2.3.2. Search for Genes Associated with the Biosynthesis of Teichoic Acids

A search for genes encoding proteins typically involved in the biosynthesis of teichoic and lipoteichoic acids (TAs and LTAs) was conducted using BLAST searches against a custom database, in a manner similar to the search for peptidoglycan-associated genes. Amino acid sequences of proteins encoded by the genes *tagA*, *tagB*, *tagC*, *tagD*, *tagE*, *tagF*, *tagG,* and *tagO* [56,57] were used to identify homologs in the genome of *Bulleidia extructa* PP_925 and other bacteria. Only a glycerol-3-phosphate cytidylyltransferase domain homologous to the TagD protein was identified in the encoded proteins of *B. extructa* PP_925 as well as in *B. extructa* W1219^T^. The corresponding protein is located within the locus 00310–00350, involved in the biosynthesis of surface polysaccharides. The presence of the phosphotransferase gene 00330 within this locus suggests that negative charges may be incorporated into the biosynthetic product. This modification could allow the product to functionally complement tag-synthesized teichoic acids. In contrast, a substantial proportion of representatives of other genera within the families *Coprobacillaceae* and *Erysipelotrichaceae* (21 out of 43 analyzed genomes) encoded nearly the complete set of tag proteins.

#### 2.3.3. Tight Adherence and Competence Systems

**Figure 11:**
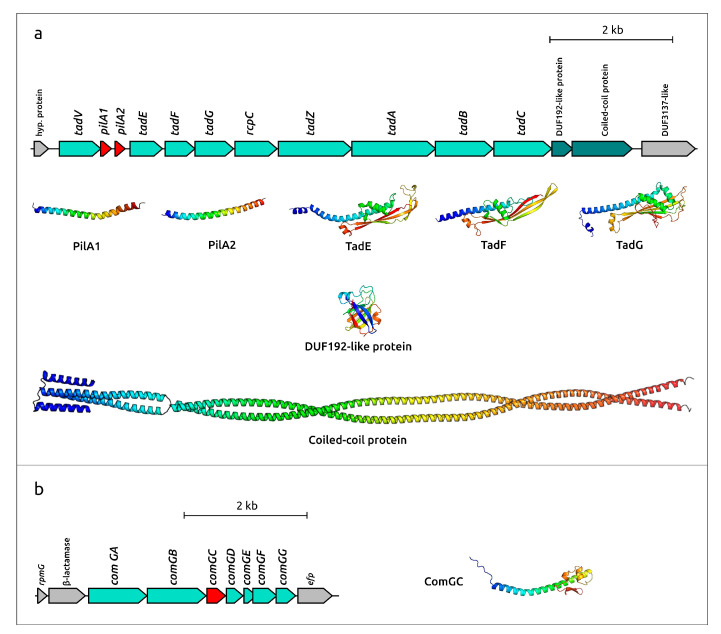
(**a**) Genetic map of the *tad* locus identified in the genome of *Bulleidia extructa* PP_925 and AlphaFold 3 (AF3) models. In the AF3 models, monomers are visualized with a blue-to-red gradient, where blue represents the N-terminus and red represents the C-terminus. (**b**) Genetic map of the *comG* locus and AF3 model of ComGC protein. Gene annotations and predicted functions in the maps are indicated by labels and the legend.

Annotation of the genome of *B. extructa* PP_925, performed with different annotation pipelines and complemented by HHblits and BLAST searches, identified two loci encoding pilin-like proteins. Detailed analysis of the genetic context of these loci suggested that one of them belongs to the tight adherence system (*tad* operon), while the other is part of the competence system (*comG* operon) (Figure 11).

*Tad* operons show noticeable diversity among Gram-positive bacteria [58,59]. The *tad* operon of *Bulleidia extructa* PP_925 displays several important features in its organization and composition, including a unique arrangement of the constituent genes. The operon in *B. extructa* PP_925 apparently comprises 13 genes. It begins with the prepilin peptidase *tadV* gene, followed by two genes, *pilA1* and *pilA2*, encoding the major pilin subunits (Figure 11a). As in other Tad pilins [60], structural models of PilA1 and PilA2 predict the absence of the C-terminal globular β-sheet domain (for comparison, see AF3 models of PilA1 and PilA2 and the model of the competence system major pilin ComGC in Figure 11a,b). Predicted minor pilins TadE and TadF, as modeled with AF3, share the same basic structural architecture but possess additional C-terminal domains compared to the major pilins. TadG is characterized by an even larger C-terminal domain, although it retains a structural organization similar to that of TadE and TadF.

A BLAST search of the amino acid sequences of *tad* operon proteins against NCBI and custom databases identified homologous proteins encoded within a similar locus (pairwise identity > 90%) in the genome of *B. extructa* W1219^T^. No homologs were found in closely related genomes of *Solobacterium*, *Lactimicrobium*, *Galactobacillus,* and *Stecheria*, except for PilA1. Numerous homologs of the conserved TadA ATPase were identified across different *Erysipelotrichia* bacteria. However, many of them were less similar to the sequences from *Bulleidia* than to homologs from other, more distantly related groups, including *Helcococcus ovis* and members of *Clostridia*. This suggests that the *tad* locus in *B. extructa* was acquired horizontally.

Functions of eleven gene products of the *tad* operon can be predicted by HMM-based searches or AF3 modeling and comparison with published structures [61,62,63]. Two genes, however, located at the 3′ end of the operon, show no clear sequence or structural similarity to functionally characterized Tad-system proteins. Nevertheless, these genes are consistently found adjacent to Tad-related genes in *B. extructa* W1219^T^, in two metagenomic sequences classified by GTDB as *Erysipelotrichales*, and in three complete genomes of *Helcococcus ovis*. The penultimate gene encodes a protein of 112 amino acids similar to DUF192 domain-containing proteins (Pfam entry PF02643). Its predicted structure (Figure 11a) closely resembles that of a “hypothetical signal peptide protein” or “putative transcription regulator” from *Sinorhizobium meliloti* (PDB code 3pjy, DALI Z-score 15.8) and of a “protein of unknown function” from *Novosphingobium aromaticivorans* (PDB code 3m7a, DALI Z-score 14.5). It also shows weaker similarity to structures such as the “putative exported protein” from *Burkholderia pseudomallei* (PDB code 6ozd, DALI Z-score 8.8). The last gene of the operon encodes a protein predicted to have a coiled-coil structure (Figure 11a). HHpred analysis revealed a strong similarity (>99% probability) to the chromosome partitioning protein Smc from *Bacillus subtilis* (PDB code 5nnv [64]) and to α-helical regions of several eukaryotic proteins, including dynein. However, DALI searches using the predicted structure did not return close structural homologs. These two proteins may play important and unique roles in the functioning of the Tad system in *B. extructa* PP_925.

Another genomic locus associated with the formation of type IV pili corresponds to the competence operon *comG*. This operon is flanked upstream by a gene encoding a β-lactamase-like protein and downstream by the elongation factor *efp*. BLAST searches using the predicted sequences of ComGA-ComGG identified homologous loci in the genomes of *Solobacterium moorei* DSM 2297, *Bulleidia* sp. zg-1006, and other *Erysipelotrichaceae* and *Erysipelotrichales*, suggesting a vertical origin of the locus. Interestingly, the β-lactamase-like gene has its closest homologs not only in *Bulleidia* sp. zg-1006, *S. moorei* DSM 2297, and other *Erysipelotrichales*, but also in several strains of *Fusobacterium* and other anaerobic bacteria, suggesting horizontal gene exchange among evolutionarily distant organisms inhabiting the same ecological niche.

#### 2.3.4. Anti-Phage Defense Systems

**Figure 12:**
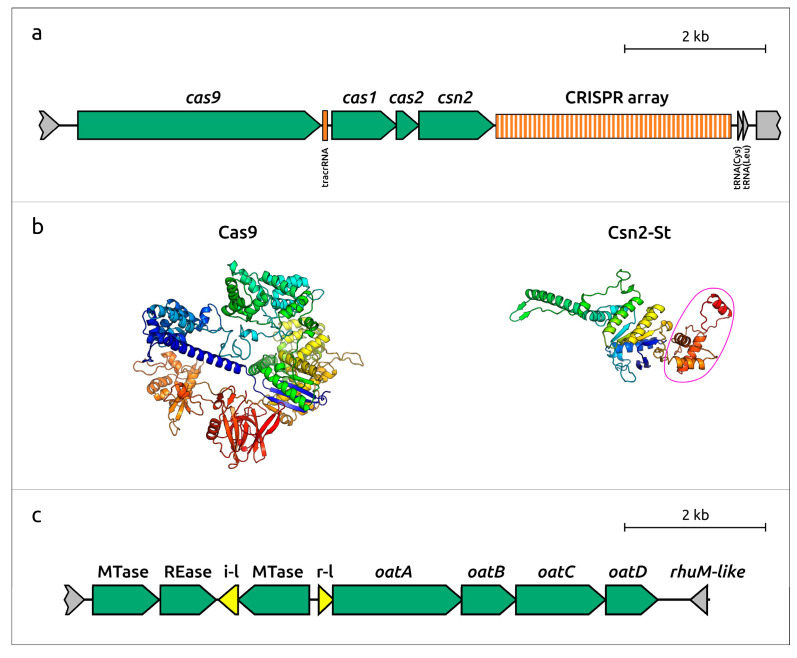
(**a**) Genetic map of the CRISPR-Cas locus identified in the genome of *Bulleidia extructa* PP_925. (**b**) AF3 models of multidomain Cas9 and Csn2-St proteins. The additional domain of Csn2-St, specific to subfamily St, is outlined in pink. The models are visualized with a blue-to-red gradient, where blue represents the N-terminus and red represents the C-terminus. (**c**) Genetic map of the RM_Gao_Qat locus. Gene annotations and predicted functions are indicated by labels and a legend. Gene names are written in italics. MTase indicates modification methyltransferase, REase indicates restriction endonuclease, i-l indicates integrase-like protein, and r-l indicates repressor-like protein. Arrows show the direction of transcription for each gene. The scale bar represents the nucleotide sequence length.

The search for anti-phage defense systems in the genome of *Bulleidia extructa* PP_925 identified several loci potentially encoding such systems. These include the following:

- Abi systems. Two single-gene defense systems of the Abi family were identified [65]. Notably, both of them are located near genes for cellular non-coding RNAs, specifically tRNA-Thr and tmRNA. This genomic arrangement suggests they may be organized as a type III toxin–antitoxin module, in which a regulatory RNA inhibits the toxin protein under normal conditions. The closest homologs were found in the complete genome of “*Bulleidia* sp. 10714-15” (identified using TBLASTN and the NCBI Core_nt database), which is likely misclassified and more plausibly represents *Solobacterium moorei*. This suggests that these genes might have been inherited vertically and subsequently lost in other related bacteria, including *Bulleidia* and *Solobacterium*. The next closest homologs of the Abi protein encoded by gene locus tag 01180 were found in genomes of two strains of *Faecalibacillus intestinalis*. Homologous sequences to the Abi protein encoded by gene locus tag 02335 were identified in *Staphylococcus gallinarum* strain X16P4, *Lacticaseibacillus paracasei* strain PC-H1, different strains of *Lactobacillus helveticus* and other bacteria.
- CRISPR-Cas system. A locus comprising genes 02725–02740 and a downstream repeat region was identified as a type II-A CRISPR-Cas system, characterized by a minimal set of core components. These include the signature effector protein Cas9 as a single multidomain nuclease, the CRISPR array, tracrRNA, Cas1 and Cas2 adaptation proteins, and the auxiliary protein Csn2 [66]. The Csn2 protein in *B. extructa* PP_925 belongs to the Csn2 subfamily St, previously described in *Bacillota*, largely in *Streptococcus* and *Enterococcus*. These proteins are longer than canonical Csn2 due to an additional C-terminal domain [67] (Figure 12a,b). The CRISPR array spacers were searched against the GenBank PHG database. No exact matches were found in phage genomes. The most similar sequences (identity up to 95%, query coverage ≤ 80%) belonged to phages infecting Gram-positive bacteria from the families *Herelleviridae*, *Salasmaviridae,* and *Vilmaviridae*; subfamily *Trabyvirinae*; and genus *Coventryvirus*, all within the class *Caudoviricetes*. BLAST searches identified the closest homologs of all four proteins of the *B. extructa* PP_925 CRISPR-Cas system in various species of the genus *Streptococcus*. However, no significant homologs were found in NCBI Core_nt genomes attributed to *Bulleidia* or closely related genera, except for limited similarity (33%) of Cas1 to a protein in “*Bulleidia* sp. 10714-15”.
- Restriction-modification (RM) type II system. A locus encoding proteins reminiscent of the type II RM system of *Haemophilus parahaemolyticus* HhaI [68,69] was identified. It includes three genes: two encoding DNA (cytosine-5-)-methyltransferases (locus tags 04715 and 04730) and one encoding a restriction endonuclease (locus tag 04720) (Figure 12c). The protein encoded by gene 04725, located between the restrictase (locus tag 04720) and the second methyltransferase (locus tag 04730), showed no significant similarity (>90% probability) in HHpred searches. However, AF modeling and subsequent DALI analysis revealed structural similarity to tyrosine recombinases, including the site-specific integron recombinase IntI4 from *Vibrio cholerae* (Z-score 7.3) and phage integrases. The gene appears to be degraded, since the encoded protein is at least twice as small as functional integrases. This suggests that the second methyltransferase may have arisen through recombination and that gene 04725 encodes a degraded integrase-like protein. Notably, the orientation of genes 04725 and 04730 is opposite to that of the rest of the RM locus. TBLASTN searches identified close homologs (up to 99% pairwise identity) of all these proteins in genomes of *Campylobacter concisus*, *Fusobacterium animalis*, *Streptobacillus moniliformis*, *Fusobacterium pseudoperiodonticum*, *Haemophilus haemolyticus,* and *Metamycoplasma arthritidis*. No *Erysipelotrichia* representatives were among the first 100 hits.
- Gao_Qat system. A complete Gao_Qat system was identified, comprising the four characteristic proteins QatA, QatB, QatC, and QatD encoded by genes 04740, 04745, 04750, and 04755. This locus is located next to the RM type II system, separated by a small gene encoding a repressor similar to phage repressors and to the competence regulator ComR from *Streptococcus suis* (HHpred probability > 98%) [70]. Adjacent to this region, a gene was found encoding a protein with strong similarity to virulence protein RhuM (HHpred probability 99.05%). However, the encoded protein is three times smaller than the canonical RhuM protein encoded within the SPI-3 pathogenicity island of *Salmonella typhimurium* [71], suggesting that it may represent a degraded copy. This gene is unlikely to be part of a mobile genetic element, since BLAST searches revealed different patterns of homology compared to the RM and Gao_Qat proteins. Searches of genes surrounding the RM and Gao_Qat loci revealed other distinct homologs not shared with these systems.

In addition, the genome of *B. extructa* PP_925 encodes the Wadjet system, which provides resistance to plasmid transformation [72,73]. This locus includes all four characteristic genes, *jetA*, *jetB*, *jetC*, and *jetD* (locus tags 01950-01965).

#### 2.3.5. Search for Prophage Regions and Phage-Derived Sequences

Search for prophage regions, conducted using PHASTEST and Phigaro, did not find prophages in the genome of *B. extructa* PP_925. Furthermore, analysis of the results of the HHblits search involving all predicted proteins encoded in the genome did not find the sequences that could represent the phage major capsid protein of *Duplodnaviria* (HK-97) or other viruses, portal protein, or terminase large subunit. The HHblits search (probability threshold 90%) found sequences similar to phage and other integrases and recombinases, which could belong to other mobile elements or represent non-functional remnants of phages or other mobile elements.

#### 2.3.6. Search for Antibiotic Resistance Genes

In the genome of *Bulleidia extructa* PP_925, we identified a gene encoding an aminoglycoside N(3)-acetyltransferase (AAC(3)) (locus tag 02460). BLASTp analysis showed that its closest homologs (up to 89.6% PI) occur in multispecies proteins annotated as *Bacillati* and in closely related *Erysipelotrichia*, including *Solobacterium* spp., indicating possible lineage-level conservation rather than recent acquisition from distant taxa. AAC(3) enzymes acetylate the 3-amino group of 2-deoxystreptamine aminoglycosides and are commonly associated with reduced susceptibility to agents such as gentamicin and related compounds, in agreement with experimental data obtained in this study; however, the substrate spectrum is variant-dependent [74,75].

A search for antibiotic resistance genes (ARGs) using the Resistance Gene Identifier (RGI) server identified only four *vanY*-like vancomycin resistance genes, potentially associated with the *vanB*, *vanF,* and *vanG* gene clusters. However, analysis of the genomic context of these *vanY*-like genes did not reveal the presence of the typical additional genes that constitute the *vanB* [76], *vanF* [77], *vanG* [78], or *vanA* [79] clusters. Furthermore, AMRFinderPlus analysis did not detect ARGs.

A BLAST search of the 1335 predicted proteins against the latest version of the CARD database identified 110 proteins associated with antibiotic resistance that are homologous to *B. extructa* PP_925 proteins. The best hits were distributed as follows: 15 proteins each from *Enterococcus* spp. and *Streptomyces* spp.; 10 from *Staphylococcus aureus*; 9 from *Neisseria gonorrhoeae*; 8 each from *Escherichia coli*, *Streptococcus* spp., and *Paenibacillus* spp.; 7 from *Riemerella anatipestifer*; 4 each from *Bacillus licheniformis*, *Clostridioides difficile,* and *Desulfitobacterium hafniense*; 2 each from *Acinetobacter baumannii*, *Bifidobacterium adolescentis,* and *Pseudomonas aeruginosa*; and 12 from other organisms. No proteins from *Erysipelotrichia* were present among the top hits. This scarcity of matches may reflect a gap in current knowledge of the molecular mechanisms of drug resistance in *Erysipelotrichiaceae*. It also likely reflects the bias in existing datasets toward widespread pathogens. HHblits searches additionally identified 32 proteins with similarity to β-lactamases and 161 proteins with similarity to diverse bacterial proteins that could potentially be associated with antibiotic resistance, highlighting the need for experimental studies to clarify the functional significance of these candidate ARGs. However, this may also reflect the susceptibility of *B. extructa* PP_925 to antibiotics observed in our experiments.

### 2.4. Search for Virulence Factors

BLASTP searches against the Virulence Factor Database (VFDB) core dataset, which includes representative genes associated with experimentally verified virulence factors (https://www.mgc.ac.cn/VFs/ accessed 20 August 2025), identified similarities between several hundred proteins of *Bulleidia extructa* PP_925 and the VFDB core set. In total, 230 predicted proteins matched with an E-value cut-off of 10^−5^. However, such sequence-level matches cannot be directly interpreted as evidence of virulence-related functions without experimental validation or more detailed in silico analysis. This is particularly important since several proteins within the VFDB core set are multifunctional and not always directly associated with virulence. Examples include elongation factor Tu in *Francisella tularensis*, which interacts with host nucleolin [80]; the nucleoside ABC transporter substrate-binding protein BmpD in *Borrelia burgdorferi*; and the DNA repair protein RecN in *Neisseria meningitidis*.

Bacterial pathogenicity typically depends on several core strategies: adherence to host tissues, evasion or modulation of host immune responses, secretion of exoenzymes that promote tissue invasion and nutrient acquisition, and stress-survival mechanisms that enable persistence and regulation of virulence expression [81,82,83,84]. The search revealed multiple *B. extructa* PP_925 proteins homologous to experimentally verified factors from these functional categories. Representative homologs of virulence factors in *B. extructa* PP_925 include the following:

- Stress survival: Clp ATP-binding chain C (ClpC, VF0072; *Listeria monocytogenes* EGD-e).
- Exoenzymes: Hyaluronidase (HysA, VF0146; *Streptococcus pneumoniae* TIGR4).
- Adherence: *Listeria* adhesion protein Lap (Lap, VF0444; *L. monocytogenes* EGD-e); chaperonin GroEL (GroEL, VF0594; *Clostridium difficile* 630); Tad pilus protein (CpaF/TadA, VF0612; *Vibrio vulnificus* CMCP6) encoded in the aforementioned *tad* locus; fibronectin-binding protein (FbpA, VF0349; *L. monocytogenes* EGD-e); collagen adhesin precursor (CNA, VF0005; *Staphylococcus aureus* MW2); laminin-binding surface protein (Lmb, VF0275; *Streptococcus agalactiae* NEM316).
- Immune modulation: Cap8D (VF0003; *S. aureus* MW2); phosphomannomutase ManB/YhxB (VF0044; *Haemophilus influenzae* Rd KW20); undecaprenyl diphosphate synthase CpsA/UppS (VF0361; *Enterococcus faecalis* V583); LCP family protein GBS_RS06610 (VF0274; *S. agalactiae* NEM316); Bcs1′ (VF0043; *H. influenzae* 1007); CapA (VF0141; *Bacillus anthracis*); aminotransferase Cj1437c (VF0323; *Campylobacter jejuni* NCTC 11168); HasC (VF0244; *Streptococcus pyogenes* M1 GAS). Interestingly, the LCP family protein and a capsular polysaccharide synthesis-like protein are encoded in a genomic region apparently responsible for PG modifications or exopolysaccharide synthesis.
- Exotoxins: Hemolysin B (HlyB/HlyA, VF0225; Escherichia coli CFT073); Toxin A (TcdA, VF0376; *C. difficile* 630).

BLAST searches also indicated that the closest homologs of 8 out of these 18 *B. extructa* PP_925 proteins were found in bacteria other than the closest relatives of *Bulleidia* (*Solobacterium moorei* and *Lactimicrobium massiliense*).

### 2.5. Pangenome Analysis

**Figure 13:**
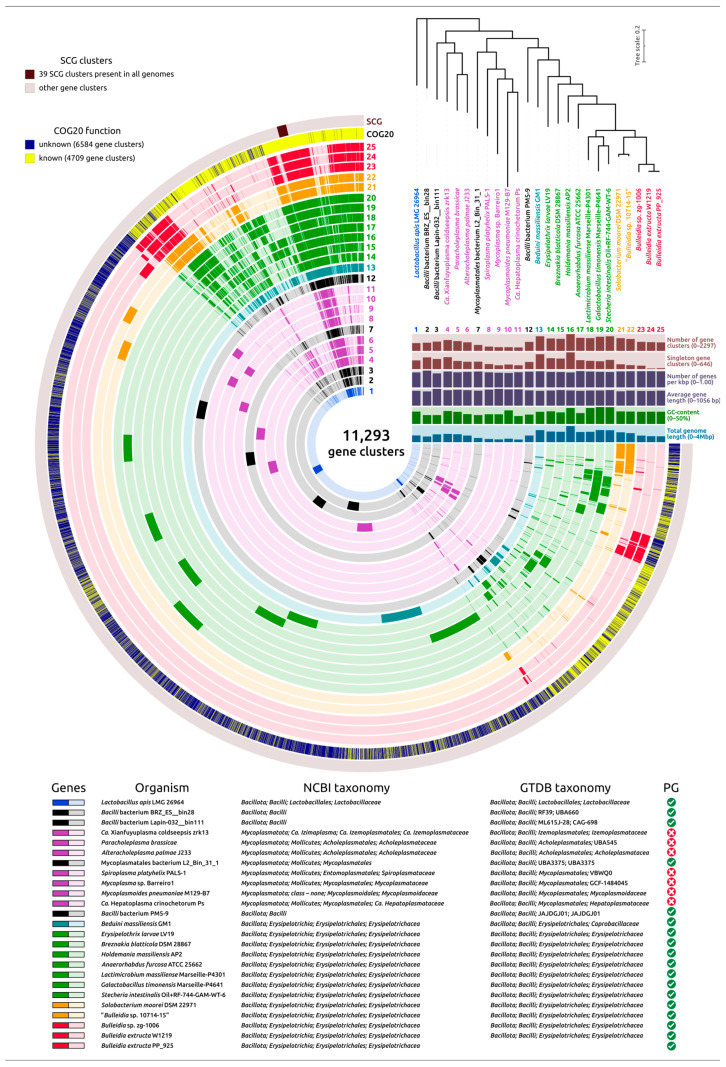
Pangenome structure of a 25-genome dataset highlighting the position of *Bulleidia extructa*. Genomes are ordered by a single-copy core gene (SCG) phylogeny (outer dendrogram), and concentric radial layers represent individual genomes aligned against the same catalog of gene clusters. Angular segments correspond to gene clusters ordered by their frequency across the dataset; filled, saturated ticks denote the presence of a cluster in a given genome, whereas pale gaps indicate absence. Outermost rings annotate cluster properties (COG20 functional category and single-copy core membership) and are colored according to the legend. The phylogenetic tree is based on concatenated SCG sequences. *Lactobacillus apis* LMG 26964 was used as the outgroup. The scale bar indicates 0.2 substitutions per site; bootstrap support values were ≥83%.

An *anvi’o* pangenome analysis of 25 genomes spanning the *Erysipelotrichales*, *Lactobacillales*, *Mycoplasmatales*, and atypical *Bacillales* (Figure 13) revealed a total of 11,293 gene clusters, of which only 39 single-copy core gene clusters (SCGs) were present in all genomes. This small universal core reflects the deep evolutionary divergence across the dataset and highlights the high degree of lineage-specific adaptation. Within the *Bulleidia* group, 66 gene clusters were found to be unique to the genus (*B. extructa* PP_925, *B. extructa* W1219^T^, and *Bulleidia* sp. zg1006), while 93 clusters were shared exclusively between the two *B. extructa* strains, suggesting significant lineage-specific lateral gene flow. The overall functional composition of the pan-genome was skewed toward poorly annotated regions: approximately 58% of clusters could not be assigned to known COG20 functional categories, forming “genomic dark matter” often found in lineage-specific arcs in Figure 13. Nevertheless, *Bulleidia extructa* stood out for its relatively high fraction of annotated functions compared with both larger and smaller genomes. Despite its minimal size, the *B. extructa* genome has a surprisingly high fraction of functionally characterized genes relative to many other bacteria with reduced genomes. Only roughly 24–27% of its genes are of unknown function, meaning ~73–76% could be assigned to known COG functional categories in annotations. This proportion of “unknown” genes is markedly lower than that in larger relatives or other minimal bacteria. For example, the 3.8-Mbp genome of *Holdemania massiliensis* (another *Erysipelotrichaceae* member) has about 34% of genes with no known function in COG analysis, the genome of *Mycoplasmoides pneumoniae* M129-B7 encodes 35% or more such proteins (despite their importance and intensive study as an important pathogen), and *Mycoplasma* sp. Barreiro1 has 30% of such genes, as was revealed by the present analysis. This low content of functionally uncharacterized genes can be a consequence of the specifics of *Bulleidia’s* gene repertoire: it appears to have retained mostly well-characterized core functions and shed dispensable or novel gene families above the limit necessary for its lifestyle. Turning to singleton gene clusters, *B. extructa* PP_925 presented only 38, fewer than any other genome in the dataset (e.g., *M. pneumoniae* M129-B7 has 161, *Erysipelothrix larvae* LV19 has 309, *H. massiliensis* AP2 has 609). Given the close evolutionary relationship between PP_925 and W1219^T^, this low singleton count plausibly reflects their recent common ancestry and limited divergence. Finally, average gene lengths and coding densities varied by only ~10–20% across genomes, implying that genome reduction occurred predominantly through gene loss, rather than through compaction of gene or intergenic regions.

To further interpret these genomic patterns, a phylogenetic analysis was performed using the 39 single-copy core genes (SCGs) identified using the 30% identity threshold (other parameters are clarified in Section 4). A concatenated SCG tree was constructed, with *Lactobacillus apis* included as an outgroup to root the topology (Figure 13). The evolutionary history reflected in the topology of the SCGs tree follows that of the GTDB tree (Figure 5) and, to a lesser degree, the ribosomal proteins tree (Figure 6). In the SCG tree, all *Erysipelotrichales* (including *Bulleidia*) cluster together on a distinct branch, separate from the mollicutes (cell-wall-less mycoplasma-related clades) and representing a strikingly versatile group, by genomic size and content. As in other trees discussed earlier in the article, the cell-wall-less bacteria in the analysis do not form a single monophyletic group; instead, they appear in two different clades, each branching within or next to cell-wall-bearing relatives, suggesting at least two independent origins of the mollicute lifestyle. In one clade, a group of mycoplasmas is phylogenetically interwoven with peptidoglycan-containing relatives at its deepest branches; in another clade, a separate lineage of wall-less bacteria (possibly an anaeroplasma or asteroleplasma group) branches from within a big clade containing unclassified *Bacillalli* and *Erysipelotrichales*. For completeness, we note that this placement reiterates a well-established result: the class Mollicutes (the only member of the phylum Mycoplasmatota) is polyphyletic, having arisen from Firmicute (phylum Bacillota) ancestors multiple times [9]. Combining pangenome and phylogenetic results, we infer two contrasting trajectories within this clade: genome minimization (mycoplasma-like bacteria) and genome expansion/innovation. Both trajectories are present within *Erysipelotrichales*, with minimization in some lineages (*Bulleidia*) and expansion/innovation in others (e.g., *H. massiliensis*).

**Figure 14:**
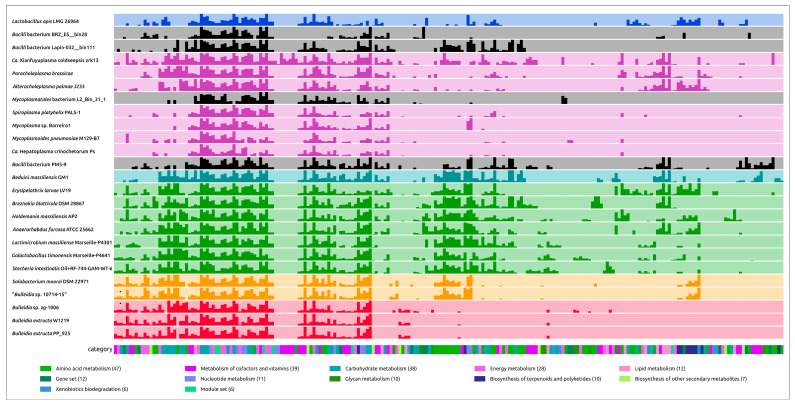
Summary metabolic potential across the genome panel (collapsed categories). Rows correspond to genomes; columns group KEGG pathways/modules into major categories. For each pathway, a colored bar encodes percent completeness (bar height), paler shades mark incomplete or missing modules, and colors in the bottom lane “category” indicate the KEGG category according to legend.

The metabolic genomic analysis of *B. extructa* PP_925, visualized in the summary heatmap of KEGG pathway comparison (Figure 14) and detailed in Supplementary Figure S15, reveals a profound reduction in anabolic and energy-generating functions, with retention of only a narrow fermentative core. At the global scale, the *Bulleidia* strains clustered with other *Erysipelotrichales*, sharing a number of common features in metabolism and, like the closest relatives of *Bulleidia extructa*, showing a reduction in metabolism. However, the *Bulleidia extructa* rows were particularly sparse. Both strains of *B. extructa* exhibit a strongly reduced metabolic repertoire compared to most other *Bacillota* analyzed. The KEGG pathway comparison highlights that *B. extructa* retains only a limited subset of central metabolic functions, with conspicuous losses in amino acid biosynthesis, carbohydrate utilization, and energy metabolism. This pattern indicates a streamlined physiology consistent with adaptation to a nutrient-rich, host-associated environment. When contrasted against other *Erysipelotrichales* (e.g., *Solobacterium*, *Holdemania*), *B. extructa* shows a shared loss of oxidative phosphorylation, the TCA cycle, and broad amino acid biosynthesis, but stands out as one of the most reduced in nucleotide and lipid pathways. In the broader comparison including mycoplasmas, the pangenome analysis (Figure 13) illustrates how *Bulleidia* retains large blocks of clusters absent in mycoplasmas, while converging with them in genome reduction and losing many biosynthetic functions.

- Carbohydrate metabolism. Despite broad gene loss, glycolysis was retained in full, providing a backbone for ATP generation. The analysis (Figure 14, Supplementary Figure S15) indicated the presence of the Embden–Meyerhof–Parnas pathway, as well as sugar transport modules, including phosphotransferase systems (PTS). The presence of the phosphotransacetylase–acetate kinase (Pta-AckA) pathway [85] enables conversion of acetyl-CoA to acetate with ATP generation via substrate-level phosphorylation. Experimental studies confirm fermentation of glucose and maltose, yielding acetate, lactate, and succinate as end-products [22]. The ability to generate ATP from acetate production places *Bulleidia* in an intermediate position: it shares fermentative minimalism with mycoplasmas, but retains additional flexibility through this ancestral pathway.
- Energy metabolism. No respiratory complexes or cytochromes were detected. TCA modules are nearly absent, leaving only isolated enzymatic fragments without cycle functionality. Thus, *B. extructa* is strictly fermentative, relying on substrate-level phosphorylation. Despite lacking respiratory complexes, the genome retains F- and V-type ATPases that can reverse-operate, hydrolyzing ATP to pump H^+^ and maintain a membrane potential.
- Amino acid metabolism. The genome lacked nearly all de novo amino acid biosynthetic pathways. Most KEGG pathways are absent or incomplete, suggesting that *B. extructa* relies heavily on exogenous sources or metabolic cross-feeding within microbial communities. By contrast, several related taxa (e.g., *Holdemanella*, *Anaerorhabdus*) still retain partial biosynthetic capacities. Notably, arginine biosynthesis genes were absent, yet *B. extructa* retains the arginine dihydrolase (ADI) pathway for catabolism, consistent with experimental evidence of arginine hydrolysis [22].
- Nucleotide metabolism. *B. extructa* lacks genes for both purine and pyrimidine biosynthesis, and a complete loss of de novo purine and pyrimidine biosynthesis for *B. extructa* W1219 was predicted earlier [11]. Instead, *Bulleidia* seemingly relies on salvage reactions: rather than synthesizing nucleotides from scratch, the bacterium recycles free bases and nucleosides imported from the environment via phosphoribosyltransferases and kinases. These salvage pathways apparently compensate for the absence of de novo routes, but enforce strict dependence on exogenous nucleotide sources.
- Lipid metabolism. Fatty acid (Fab) biosynthetic genes are absent, making *Bulleidia* auxotrophic for fatty acids. This explains its growth requirement for Tween 80 (a source of oleic acid) [22].
- Cofactor and vitamin metabolism. Most B-vitamin and cofactor pathways are absent (e.g., biotin, thiamine, riboflavin), forcing reliance on host-derived vitamins.

## 3. Discussion

The currently available data are consistent with the presence of the genus *Bulleidia, which* includes more than ten sequenced representatives. Based on 16S rRNA gene similarity, these sequences appear to fall into three tentative species-level clusters. The type species *Bulleidia extructa* is represented by strains PP_925, the type strain W1219(T), and several 16S rRNA sequences; a second putative lineage is represented by the draft genome *Bulleidia* sp. zg-1006 and a 16S rRNA sequence; and a third group currently corresponds to 16S rRNA sequences only and thus remains to be confirmed by genome-based analyses.

A complicating factor is the inconsistent classification in public databases. In NCBI GenBank several representatives of the related genus *Solobacterium* are annotated as *Bulleidia*. This reflects not only errors in deposited sequence labels but also the close relatedness of these two genera, as confirmed by phylogenetic analyses with different gene sets. Comparisons based on AAI and POCP show values of about 64% and 51% between type representatives of *Bulleidia* and *Solobacterium*. These values are close to the commonly accepted genus-level thresholds of about 60% for AAI and 50% for POCP [46,48]. Nevertheless, pangenome analysis and metabolic profiling reveal clear differences in gene content and functional potential, supporting the separation of *Bulleidia* and *Solobacterium* as distinct genera despite numerical proximity to these thresholds. Consistent with this assignment, ANI calculations confirm the close relatedness of PP_925 and the type strain W1219^T^.

In the Genome Taxonomy Database (GTDB), several closely related genera, including *Bulleidia*, *Solobacterium*, *Lactimicrobium*, *Galactobacillus,* and *Stecheria*, are placed together under the genus *Bulleidia*. This reflects the principles of GTDB taxonomy, which relies on genome-wide phylogenies constructed from a standardized set of 120 conserved marker genes (BAC120) and applies rank normalization to ensure comparable divergence levels across clades [86,87]. In our analysis, these taxa form a cluster with pairwise similarities of up to 67% AAI and 56% POCP, and phylogenomic reconstructions recover them as a monophyletic and distinct lineage within *Erysipelotrichiaceae*. The coherence of this block and its treatment in GTDB suggest that its formal delineation as a higher-order taxonomic unit might merit consideration in the future.

Phylogenetic reconstructions based on GTDB marker sets and ribosomal protein sequences, including metagenome-derived representatives, confirm the monophyly of the order *Erysipelotrichales*, but also reveal a more complex structure of the larger clade that includes cell-wall-less mycoplasma-like bacteria. This topology refines the current view of *Erysipelotrichales* evolution. In particular, it highlights the proximity of *Erysipelotrichales* to a group of small-genome (1.1–1.6 Mbp) unclassified bacteria, including one assembly erroneously annotated in NCBI as *Erysipelotrichaceae bacterium* OttesenSCG-928-M19, which in fact lies outside the family *Erysipelotrichaceae*. At greater phylogenetic distance, the analyses also indicate relatedness to the small-genome lineage *Candidatus* Caccosoma. Consistent with previous work [9], cell-wall-less mycoplasma-like bacteria are not recovered as a monophyletic group. Instead, they are distributed among separate clades, each of which contains basal branches represented by cell-wall-bearing organisms. This pattern supports the view that the loss of peptidoglycan occurred multiple times independently from peptidoglycan-containing ancestors, providing additional resolution to the evolutionary trajectory of the broader *Erysipelotrichales*–*Mollicutes* assemblage.

Within this context, the phylogenetic proximity of *Bulleidia extructa* to *Solobacterium moorei* aligns with a lifestyle typical of opportunistic pathogens. *S. moorei* is repeatedly linked to halitosis and oral pathology and is documented in wound and bloodstream infections in susceptible hosts, supporting an opportunistic profile [88,89,90]. *B. extructa*, though infrequently reported, was originally described from the oral cavity and has been documented in rare serious infections such as periprosthetic joint infection [25]. Genomic features reinforce this profile, including adhesion-associated loci such as the *tad* operon and a competence system, homologs of adherence factors, and a broad suite of anti-phage defense systems. These properties are typical of low-abundance commensals that may expand under dysbiotic conditions and contribute to opportunistic infections.

The analysis of *Bulleidia extructa* PP_925 demonstrates the recurrent theme of reductive evolution among host-associated *Bacillota*. Genome erosion in such bacteria is not accidental but reflects adaptive tuning to nutrient-rich environments. The ~1.4 Mbp genome of *B. extructa* ranks among the smallest in cell-wall-containing *Bacillota*, illustrating a balance between metabolic necessity and redundancy removed due to host provisioning [91]. Despite wide gene loss, core fermentation pathways persist, signaling selective pressure for energy extraction. Glycolysis remains intact, and the phosphotransacetylase–acetate kinase route enables anaerobic ATP generation. The arginine deiminase pathway (ADS) is notable both for its energy-yielding capacity and its buffering effect on environmental pH, particularly in oral habitats where acidification drives cariogenic processes. Elevated ADS activity has been observed in caries-free individuals compared with those with active lesions, pointing to a protective role of arginine metabolism in dental microbial communities [92]. Experimental models and clinical observations show that arginine metabolism increases ammonia production, raises pH, and suppresses acidogenic bacteria, which can limit caries development [93]. These observations support the interpretation that retention of arginine catabolism in *Bulleidia extructa*, despite its streamlined genome, reflects a conserved strategy of environmental adaptation relevant to its ecological niche. Notably, our systematic survey of antibiotic resistance determinants in PP_925 revealed only a single clearly identifiable aminoglycoside N(3)-acetyltransferase and a small number of candidate loci with distant similarity to curated ARGs, consistent with its predominantly susceptible phenotype. Given the paucity of experimentally characterized resistance determinants in *Erysipelotrichia* and the bias of existing databases toward well-studied pathogens, robust links between genotype and the antibiotic susceptibility profile of *B. extructa* will have to come from dedicated functional work, including targeted gene knockouts, expression analyses, and biochemical characterization in future studies.

Phylogenomic and pangenomic analyses place *B. extructa* among *Erysipelotrichales*, highlighting different trajectories of genome evolution within *Bacillota*. *Mycoplasmatota* independently evolved wall loss in association with a host-dependent lifestyle, whereas *B. extructa* indicates that retention of peptidoglycan remains adaptive in dynamic host-associated niches. Furthermore, in *Mycoplasmatota* such as *Mycoplasmoides pneumoniae* and related bacteria, the evolutionary trajectory is largely unidirectional, with progressive gene loss leading to extremely small genomes lacking most biosynthetic and envelope functions. In contrast, within *Erysipelotrichales,* the pattern is more variable. Some lineages, such as *Bulleidia,* show reduced genomes, while others retain comparatively large repertoires. Gene content can both contract and expand, reflecting multiple independent episodes of genome reduction [9]. Similar observations have been made for gut-associated *Bacillota,* where adaptation to host environments frequently coincides with loss of sporulation and biosynthetic capacities, but with heterogeneous outcomes across lineages [94].

The retention of a horizontally acquired *tad* locus underscores the importance of adherence systems even in highly reduced genomes since tad pili facilitate host colonization in a range of bacteria [33,95], while competence loci can enhance genetic adaptability in streamlined genomes. Presence of homologs of known adherence-associated virulence factors further supports active rather than passive host interaction [96].

Anti-phage defense mechanisms of *B. extructa* PP_925 are unusually comprehensive. Restriction-modification systems, Wadjet-like loci, and other defense systems are retained despite apparent pressures for gene loss. CRISPR spacer analysis provides possible evidence of exposure to phages, which can include SPO1-like *Herelleviridae* myoviruses and φ29-like podoviruses, yet no prophages are found, suggesting robust defense rather than frequent viral integration. However, the presence of unique gene clusters suggests ongoing horizontal gene exchange. These patterns reinforce the view that reductive evolution in *B. extructa* is highly selective and modular.

Ecologically, *B. extructa* functions as a community specialist, depending on metabolic cross-feeding while ensuring persistence through adhesion and defense. Despite not being a common pathogen, *Bulleidia* possesses a genomic profile indicative of opportunistic behavior in compromised hosts. Furthermore, ecological investigations underscore that underappreciated taxa like *Bulleidia* can significantly influence microbial community dynamics, particularly in dysbiotic environments [97].

In summary, *B. extructa* illustrates that Gram-positive bacteria can undergo substantial genome reduction while retaining structural and adaptive features. From an evolutionary perspective, *B. extructa* exemplifies how members of *Erysipelotrichia* can occupy highly specialized roles through metabolic contraction. Its streamlined genome reflects the trade-off between independence and specialization: by discarding biosynthetic versatility, it achieves tighter integration into the metabolic networks of host-associated microbial ecosystems. Future experimental validation of substrate utilization and metabolite exchange will be essential to confirm these genomic predictions and to clarify the ecological role of *B. extructa* within host-associated consortia.

## 4. Materials and Methods

### 4.1. Strain Isolation and Cultivation

Strain *Bulleidia extructa* PP_925 was isolated in March 2024 from the periodontal pocket of a 64-year-old woman (in Moscow, Russia) with periodontitis, where it was present at a concentration of ~2.0 × 10^8^ CFU/mL in the sample. A sample was collected from periodontal pockets using sterile paper points (#30; three per site) [98]. To prevent salivary contamination, the sampling area was isolated with cotton rolls. The points were inserted to the full depth of the pocket and retained for 10 s to allow for absorption. They were then immediately transferred into sterile tubes containing 1 mL of thioglycollate transport medium (HiMedia Laboratories, Mumbai, India). In the laboratory, microbiological examination was performed according to a previously described method [99]. The isolation of strictly anaerobic bacteria was carried out on Schaedler Anaerobe Agar (Oxoid, Basingstoke, UK) supplemented with 5% (*v*/*v*) defibrinated sheep blood, Anaerobe Basal Agar (Oxoid, Basingstoke, UK) with sheep blood, and Wilkins-Chalgren Anaerobe Agar (Oxoid, Basingstoke, UK) with sheep blood. Inoculation was performed using 10^−6^, 10^−7^, and 10^−8^ dilutions of the sample. Following inoculation, the plates were placed into anaerobic jars (Schütt Labortechnik GmbH, Göttingen, Germany) with an atmosphere of 85% N_2_, 10% H_2_, and 5% CO_2_, using platinum catalysts, and incubated at 37 °C for 72 to 240 h (3–10 days). After incubation, the plates were examined macroscopically. Colony morphology was assessed, and colonies of each type were enumerated. Selected colonies were then examined microscopically following Gram staining. Distinct colonies were subcultured onto fresh plates of the same media and incubated anaerobically to obtain sufficient biomass for identification and preservation. Selected strains were preserved by lyophilization. Microbial suspensions were prepared in a cryoprotectant solution (10% sucrose and 1% gelatin (*w*/*v*)), frozen, and lyophilized using an SB1 freeze dryer (Chemlab, Barnsley, UK). The lyophilized strains were stored at –80 °C. Protocol No. 13 (15 December 2022) for the study was approved by the Ethics Committee of the Medical Institute at RUDN University (Moscow, Russia).

Biochemical reactions were determined in triplicate by using the API 20A anaerobe test kit (bioMérieux, Marcy-l’Étoile, France) and Rapid ID 32A anaerobe identification kit (bioMérieux) using incubation for 48 h and 4 h, respectively. Disc-diffusion tests were carried out using Antimicrobial Susceptibility Test Discs (Bioanalyse, Ankara, Türkiye) [100,101].

### 4.2. Electron Microscopy

For transmission electron microscopy (TEM), the *Bulleidia extructa* PP_925 was grown for 72 h in a liquid medium of the following composition (g l^−1^): tryptone (Difco, Detroit, MI, USA), 10.0; papaic digest of soybean meal (BBL Phytone Peptone, BD Biosciences, Sparks, MD, USA), 5.0; yeast extract (Difco, Detroit, MI, USA), 10.0; glucose, 0.2; NaCl, 5.0; and arginine hydrochloride, 1.0; pH 6.5. To obtain a bacterial pellet, cultures were centrifuged at 8000× *g* for 3 min in 0.1 M sodium cacodylate buffer (pH 7.2; Honeywell Fluka, Buchs, Switzerland) containing 4% sucrose (PanReac AppliChem, Darmstadt, Germany), followed by three washes in the same buffer. Primary fixation was performed for 1 h at room temperature in 0.1 M sodium cacodylate buffer supplemented with 4% sucrose and 0.05% ruthenium red (Merck KGaA, Darmstadt, Germany), containing 2% paraformaldehyde (PanReac AppliChem, Darmstadt, Germany) and 2.5% glutaraldehyde (Electron Microscopy Sciences, Hatfield, PA, USA). After three washes in 0.1 M sodium cacodylate buffer, pellets were post-fixed in 1% osmium tetroxide prepared in 0.1 M sodium cacodylate buffer for 60 min at 4 °C on ice. Dehydration was carried out in ethanol (70% and 90% for 5 min each, then 100% twice for 10 min), followed by propylene oxide (Acros Organics, Geel, Belgium) for 2 × 10 min. Pellets were infiltrated with epoxy resin (Epon 812; Electron Microscopy Sciences, Hatfield, PA, USA), first with a 1:1 (*v*/*v*) mixture of resin and propylene oxide for 60 min, then with pure resin overnight, and polymerized for 24 h at 60 °C. Ultrathin sections (~90 nm) were cut on a Reichert-Jung Ultracut E ultramicrotome (C. Reichert AG, Vienna, Austria) using a 45° glass knife, mounted on 200-mesh copper grids, air-dried, and stained with 2% uranyl acetate for 5 min in the dark. Samples were imaged on a LEO 912 AB Omega transmission electron microscope (Carl Zeiss, Oberkochen, Germany) operated at 80 kV under standard conditions.

### 4.3. Genome Sequencing

Genomic DNA was extracted using the ExtractDNA Blood & Cells kit (Evrogen, Moscow, Russia) according to the manufacturer’s instructions. DNA integrity was assessed by electrophoresis in a 1.5% agarose gel, and DNA concentration was measured with the Spectra Q HS Plus kit (Raissol Bio, Moscow Region, Russia) on a Qubit 2 fluorometer (Invitrogen, Carlsbad, CA, USA). Sequencing libraries were prepared with the MGIEasy Fast PCR-FREE FS DNA Library Prep Set v2.0 (MGI, Shenzhen, China) following the manufacturer’s protocol. Libraries were pooled equimolarly to ensure uniform coverage across samples, and the pooled library was circularized using the DNBSEQ MGIEasy Dual Barcode Circularization Kit (MGI, Shenzhen, China). Whole-genome sequencing was performed on a DNBSEQ-G50 platform (MGI, Shenzhen, China), generating 150 bp paired-end reads on an FCL PE150 flow cell (MGI, Shenzhen, China).

### 4.4. Functional Annotation, Protein Structure Prediction and Analysis, Search for Antibiotic Resistance Genes and Virulence Factors

Genomes were downloaded from the NCBI GenBank databases (https://www.ncbi.nlm.nih.gov/genbank/, accessed 20 August 2025). Annotation procedures were carried out using Bakta version 1.10 [102]. Additional analyses included BLAST searches in the NCBI databases (https://blast.ncbi.nlm.nih.gov, accessed 20 August 2025) [103], searches with InterPro [104], HHpred [105] searches against the databases PDBmmCIF70, NCBI_conserved_domains, Pfam-A, and UniProt_Swiss_Prot_viral70 [105], as well as HH-suite version 3.3.0 searches [106]. Structural predictions, homology searches, and comparisons are described below.

CRISPR loci were identified with MinCED version 0.4.2 (https://github.com/ctSkennerton/minced, accessed 20 August 2025) using the “-gffFull” setting. Spacer sequences were extracted with MinCED using the “-spacers” setting. Prophage-derived regions were identified using PHASTEST version 1.0.1 [107] and Phigaro version 2.4.0 [108], both implemented in the Proksee server [109]. HH-suite version 3.3.0 searches were additionally used to screen for phage major capsid protein and large terminase subunit sequences against the databases PDBmmCIF70, NCBI_conserved_domains, Pfam-A, and UniProt_Swiss_Prot_viral70. Genetic maps were visualized using clinker [110].

All protein structures were modeled with AlphaFold 3 [111] and visualized using PyMOL version 2.5.4 (Schrödinger Inc., New York, NY, USA). Structural similarity searches were performed with the DALI server using default settings, and similarity was evaluated with the DALI Z-score [112,113].

The search for antibiotic resistance genes was performed using AMRFinderPlus version 4.2.5 [114] and Resistance Gene Identifier (RGI) version 6.0.3 in conjunction with the Comprehensive Antibiotic Resistance Database (CARD) [115] using default settings. Virulence factors were identified by BLAST+ version 2.12.0 searches against a custom database containing the VFDB core dataset [116].

### 4.5. Phylogenetic Analysis Using 16S rDNA, Ribosomal Proteins, and 120 Bacterial Marker Genes

Nucleotide sequences of 16S rRNA genes were identified using BLAST+ version 2.12.0 or extracted from annotated bacterial genomes and aligned with Geneious Prime version 2025.0.3 (Biomatters, Inc., Auckland, New Zealand) and MAFFT version 7.48 [117] applying the L-INS-i algorithm with default settings. Ribosomal protein amino acid sequences were identified using the UBCG2 tool [118], aligned with MAFFT, and concatenated in Geneious Prime. Concatenated alignments of 120 bacterial marker genes were obtained using GTDB-Tk version 2.4.0 [119] and GTDB Release 220, applying the classify workflow with default settings.

All alignments were used for phylogenetic reconstruction with IQ-TREE version 2.4.0 [120], using automatic best-fit substitution model selection and bootstrap analysis with 1000 replicates (command line parameters: -m TEST -ninit 1000 -bb 1000). Preliminary analyses were performed with FastTree version 2.1.11 [121] under default settings. Genome completeness and contamination were estimated with CheckM2 version 1.1.0 [122] under default settings.

### 4.6. ANI, AAI, and POCP Calculations

Average nucleotide identity (ANI) was calculated with orthoANIu version 1.40 [123] using default settings and fasta files downloaded from NCBI. Clustering was performed using the UPGMA hierarchical clustering method. Average amino acid identity (AAI) calculations and clustering were performed with the EzAAI calculator version 1.2.3 [124] using default settings and genomic fasta sequences. The percentage of conserved proteins (POCP) was calculated with POCP-nf version 2.3.6 [125] under default settings using protein sequences predicted by Bakta.

### 4.7. Anvi’o Pangenome and Metabolism Analysis

Pangenome and metabolic analyses were conducted with anvi’o version 8 [126]. The pangenome analysis was performed with the program anvi-pan-genome following the workflow described in [127] and in online tutorials (https://merenlab.org/2016/11/08/pangenomics-v2, accessed 20 August 2025). Phylogenetic analysis based on concatenated alignments of single-copy core genes (SCGs) was used to order layers in the anvi’o visualization schemes. Programs and settings included anvi-get-sequences-for-gene-clusters, trimal -gt 0.50, and iqtree -s -m WAG -bb 1000.

KEGG functions and metabolic pathways [128] were annotated with anvi-run-kegg-kofams. The completeness of each KEGG pathway in every genome was assessed with anvi-estimate-metabolism, which uses previously assigned KEGG ortholog (KO) annotations. Pathway completeness matrices were visualized as heatmaps using anvi-matrix-to-newick, anvi-interactive, and other anvi’o tools following the tutorials (https://merenlab.org/tutorials/infant-gut/, accessed 20 August 2025) [129].

## 5. Conclusions

The genome of *Bulleidia extructa* PP_925 is compatible with pronounced reduction under retention of a peptidoglycan-containing cell wall. In contrast to *Mycoplasmatota* that have lost peptidoglycan, this lineage shows that streamlining can proceed without envelope loss. Loss of most amino acid, nucleotide, fatty acid, and cofactor biosynthesis pathways indicates a strongly restricted metabolic repertoire and suggests dependence on salvage pathways and metabolic support from surrounding microbiota.

A complete *tad* locus and a competence *comG* system indicate that functions related to adhesion, colonization, and genetic plasticity are preferentially retained. The defense repertoire combines CRISPR-Cas II-A with Csn2-St, a type II restriction–modification system adjacent to Gao_Qat-like elements, and the Wadjet module in a genome lacking detectable prophages. CRISPR spacer content indicates recurrent exposure to *Bacillota* phages, particularly *Herelleviridae* and φ29-like groups. Taken together, these features are consistent with a predominant role of active immunity rather than prophage-mediated exclusion, although this remains a working hypothesis requiring experimental validation.

Pangenome analysis reveals a very small core and few strain-specific genes, consistent with recent shared ancestry and functional focusing, whereas lineage-specific modules related to adhesion and defense underscore adaptive flexibility. Phylogenomic placement within *Erysipelotrichales* and the presence of multiple *Bulleidia* species accord with current evidence for mollicute polyphyly. Overall, *B. extructa* PP_925 represents a reductive strategy that maintains envelope integrity, host-interaction systems, and multiple defense modules while compressing biosynthetic capacity. It is further hypothesized that this genome organization may contribute to opportunistic behavior, a possibility that should be examined in future ecological and clinical studies.

## Figures and Tables

**Table 1:** Analyzed proteins involved in peptidoglycan biosynthesis.

Protein Name	Function
Alr	Alanine racemase catalyzes the interconversion of L-alanine to D-alanine, providing the key substrate for the formation of the D-Ala-D-Ala dipeptide required in peptidoglycan precursors.
Ddl	D-alanine-D-alanine ligase synthesizes the D-Ala-D-Ala dipeptide that is incorporated into the stem peptide of peptidoglycan.
FemA and FemB	Add glycine residues in the formation of interpeptide bridges in certain Gram-positive bacteria.
MurA	UDP-N-acetylglucosamine 1-carboxyvinyltransferase, initiates the pathway by transferring an enolpyruvyl group to UDP-N-acetylglucosamine.
MurB	UDP-N-acetylenolpyruvylglucosamine reductase reduces the product of MurA to form UDP-N-acetylmuramic acid.
MurC to MurF	Sequentially ligate L-alanine (MurC), D-glutamate (MurD), meso-diaminopimelic acid or L-lysine (MurE), and the D-Ala-D-Ala dipeptide (MurF) to the UDP-MurNAc backbone.
MraY	Phospho-N-acetylmuramoyl-pentapeptide-transferase, transfers peptidoglycan precursor onto the lipid carrier to form lipid I
MurG	UDP-N-acetylglucosamine:MurNAc-pentapeptide transferase, transfers N-acetylglucosamine to form lipid I and subsequently lipid II, the membrane-bound peptidoglycan precursor.
MurT	Amidates the D-glutamate residue of lipid II in many Gram-positive bacteria

## Data Availability

All relevant data are available within this article and its Supplementary Materials. The *Bulleidia extructa* PP_925 genome sequence is deposited in NCBI GenBank under accession number JBMPNY000000000.1.

## Supplementary Materials

The following supporting information can be downloaded at: https://www.mdpi.com/article/10.3390/ijms27010448/s1.

## Abbreviations

The following abbreviations are used in this manuscript:

aa	amino acid residues
AAI	average amino acid identity
ANI	average nucleotide identity
ADS	arginine deiminase pathway
ARG	antibiotic resistance gene
bp	nucleotide base pairs
CW	cell wall
kbp	1 thousand nucleotide base pairs
LTA	lipoteichoic acids
Mbp	1 million nucleotide base pairs
MGE	mobile genetic element
PG	peptidoglycan
POCP	percentage of conserved proteins
RM	restriction-modification
TA	teichoic acid
